# An In Vitro Functional Characterization of the Cholesterol-Transforming *Blautia hominis* Strain HA2291 Isolated from the Human Gut

**DOI:** 10.3390/nu18060882

**Published:** 2026-03-10

**Authors:** Warren Chanda, He Jiang, Shuang-Jiang Liu

**Affiliations:** 1 State Key Laboratory of Microbial Technology, Shandong University, Qingdao 266237, China; d2021077@mail.sdu.edu.cn; 2 Pathology and Microbiology Department, School of Medicine and Health Sciences, Mulungushi University, P.O. Box 60009, Livingstone 10101, Zambia; 3 State Key Laboratory of Microbial Resources, and Environmental Microbiology Research Center (EMRC), Institute of Microbiology, Chinese Academy of Sciences, Beijing 100101, China

**Keywords:** gut microbiota, *Blautia hominis* HA2291, cholesterol, cholest-4-en-3-one, epicholestanol, probiotic, hypercholesterolemia, sterol carrier protein 2 like (SCP2-like) protein

## Abstract

**Background/Objectives**: Cholesterol is an essential lipid required for membrane structure and normal physiological functions. However, dysregulation of cholesterol homeostasis, manifesting as hypercholesterolemia, can precipitate a range of metabolic and cardiovascular diseases. *Blautia* species are important gut commensals, but their role in cholesterol metabolism remains poorly defined. **Methods**: A total of 63 *Blautia* strains isolated from human fecal samples were screened for cholesterol conversion using the o-phthalaldehyde colorimetric assay in cholesterol-containing media with or without oxgall. Cholesterol removal by live and heat-inactivated cells was compared. Metabolomic, transcriptomic, and proteomic analyses were employed to investigate molecular mechanisms and involved genes. **Results**: Nine strains significantly lowered cholesterol levels (live cells: 31–78%; heat-inactivated cells: 8–64%), with the *B. hominis* strain HA2291, the *Blautia* sp. strain HA3515, and the *B. coccoides* strain HA4419 showing the strongest activity. Oxgall increased cholesterol removal by live cells to 74–83%, indicating bile-tolerant metabolism activity. Metabolomic profiling revealed that *B. hominis* HA2291 transformed cholesterol into cholest-4-en-3-one and epicholestanol. An SCP2-like protein, RS03310, was identified as a candidate cholesterol-interacting factor; its recombinant form catalyzed measurable NAD^+^-dependent cholesterol oxidation in vitro. **Conclusions**: *Blautia hominis* HA2291 may employ multiple in vitro strategies for cholesterol-lowering, including cell-surface adsorption (heat-inactivated cells), bile-enhanced removal (oxgall effect), and enzymatic transformation, with the gene RS03310 implicated as the main contributor. These findings provide in vitro mechanistic insights into *Blautia*-mediated cholesterol metabolism, highlight RS03310 as a candidate gene associated with cholesterol biotransformation, and advance our understanding of the potential role of *Blautia* in host cholesterol homeostasis.

## 1. Introduction

Hypercholesterolemia, defined as elevated cholesterol levels in blood, is a major modifiable risk factor for cardiovascular diseases (CVDs) such as atherosclerosis, coronary heart disease, and stroke, which remain leading causes of mortality globally [1]. Excess cholesterol accumulation in macrophages within the arterial intima promotes foam cell formation and inflammatory cascades, accelerating atherogenesis and elevating CVD risk [2]. Current lipid-lowering therapies such as statins and ezetimibe effectively inhibit hepatic cholesterol synthesis and intestinal absorption, respectively [3]. However, their long-term use is often limited by adverse effects, including musculoskeletal symptoms, increased diabetes risk, and elevated rates of hemorrhagic stroke in certain patient populations [4,5,6]. These limitations have prompted increasing interest in alternative or complementary approaches to cholesterol management [7].

As a complex ecosystem, the gut microbiota plays a key role in regulating host physiological functions, especially metabolic health [8]. The gut microbiota modulate cholesterol metabolism through a variety of mechanisms highlighting a regulatory role for microbial communities in cholesterol homeostasis [9,10]. Bile salt hydrolase (BSH) produced by gut bacteria deconjugates bile acids into free forms that are more readily excreted in feces. This process drives the liver to compensate by utilizing cholesterol for new bile acid synthesis, resulting in lowered body cholesterol [11]. Certain bacteria, such as *Bifidobacterium bifidum* PRL2010, can directly assimilate cholesterol and consequently reduce plasma total cholesterol levels in mice [12]. In addition, gut bacteria such as *Eubacterium coprostanoligenes*, *Bacteroides dorei*, *Eubacterium* sp. and certain *Clostridium* cluster IV species can convert cholesterol into non-absorbable sterols like coprostanol, which promotes its fecal elimination [13]. Beyond direct actions, microbial fermentation generates short-chain fatty acids (SCFAs), which have been reported to suppress hepatic cholesterol synthesis, promote bile acid formation, and enhance cholesterol efflux [14]. Collectively, these findings position the gut microbiota as important modulators of host cholesterol flux.

*Blautia*, a recently reclassified genus of gut commensals, has been associated with various aspects of metabolic health [15]. Several studies have reported reduced *Blautia* abundance in individuals with obesity and metabolic syndrome. For example, Benítez-Páez et al. observed depletion of *Blautia* species in obese children with insulin resistance and noted associations with elevated fecal inflammatory markers (IFN-γ, TNF-α, MCP-1) [16]. Similarly, Ozato et al. reported an inverse association between *Blautia* abundance and visceral fat area across sexes based on metagenomic analysis [17]. However, these findings were correlative and did not establish underlying molecular mechanisms. In animal models, *Blautia* enrichment has been shown to mitigate obesity and metabolic disorders [18]. Conversely, Kashtanova et al. linked higher *Blautia* levels with impaired glucose metabolism [19]. Our systematic review further highlighted this complex phenomenon, suggesting species/strain-specific associations [20]. Collectively, these findings suggest that *Blautia* may represent a promising probiotic candidate, although mechanistic uncertainties remain.

Although emerging evidence suggests that *Blautia* may influence lipid metabolism and body weight regulation [15,21,22,23], the systematic review study highlighted both positive and negative associations of *Blautia* abundance with obesity, emphasizing species- and strain-specific effects [20]. Importantly, despite these associations, the genetic and molecular mechanisms underlying *Blautia*-mediated cholesterol modulation remain largely undefined. To address this knowledge gap, we isolated and identified *Blautia* strains with cholesterol-lowering potential from healthy human feces, validated their cholesterol removal capacity using in vitro screening, and performed transcriptomic sequencing to analyze gene expression profiles in *Blautia hominis* strain during cholesterol exposure. This integrated approach aimed to identify candidate genes potentially involved in cholesterol metabolism. We demonstrate that the *Blautia hominis* HA2291 strain can incorporate and metabolize exogenous cholesterol into coprostanol stereoisomer under laboratory conditions. Together, these results provide functional insights into the cholesterol-modulating capacity of *Blautia* and contribute to a clearer understanding of its potential role in host cholesterol homeostasis.

## 2. Materials and Methods

### 2.1. Fecal Samples Preparation and Isolation of Blautia Strains

Fresh fecal samples were collected from 4 consenting healthy donors who had not used antibiotics for at least three months, following the Liu et al. [24] method, and were processed immediately. The 3-month antibiotic exclusion criterion was applied to increase the likelihood that donors harbored relatively stable and representative *Blautia* populations, thereby minimizing recent antibiotic-induced perturbations of the gut microbiota, which are known to persist for weeks to months [25,26,27]. Approximately 1 g of feces was suspended in 10 mL anoxic phosphate-buffered saline (PBS; 0.01 M, pH 7.4, Solarbio Science & Technology, Beijing, China) and filtered through 40 µm cell sieves (FALCON, Corning (Shanghai) Company, Shanghai, China). The filtrate was centrifuged at 860× *g* for 5 min, washed three times with PBS, and serially diluted (10^−1^–10^−10^). Two hundred microliters of dilutions (10^−7^–10^−10^) were spread on modified Gifu Anaerobic Medium (mGAM) agar (Hope Bio-Technology, Qingdao, China) supplemented with 0.5 g/L each of various carbohydrates (D-mannose, D-fructose, D-galactose, D-trehalose, D-cellobiose, inulin, palatinose, L-rhamnose) (Shanghai Macklin Biochemical Co., Ltd., Shanghai, China), amino acids (L-arginine, L-cysteine, 0.3 g/L L-tryptophan) (Aladdin Scientific, Shanghai, China), 5% clarified rumen fluid (Beijing Jingrui Baikang Biotechnology Co., Ltd., Beijing, China), 2.4 g/L sodium acetate and 2 g/L and sodium hydrogen carbonate (Sinopharm Chemical Reagent Co., Ltd., Shanghai, China), 0.5% hemin and 0.1% resazurin (Aladdin Scientific, Shanghai, China), 5% sheep blood (Hongquan Biotechnology, Guangzhou, China), 0.5% vitamin K1, 0.1% vitamin and mineral solutions (Coolaber Science & Technology, Beijing, China), and 15 g/L agar (Solarbio Science & Technology, Beijing, China). Plates were incubated anaerobically at 37 °C for 2–9 days, and single colonies were picked on days 3, 6, and 9 [28].

Small gray-white, convex, shiny colonies with γ-hemolysis (non-hemolytic) were picked and transferred by streaking onto mGAM agar for purification. The DNAs of single colonies were extracted using alkaline lysis method and served as the PCR template for amplification of the 16S rRNA gene using universal primers 27F (5′-AGAGTTTGATCCTGGCTCAG-3′) and 1492R (5′-GGTTACCTTGTTACGACTT-3′). PCR cycling was: 94 °C 3 min; 30 cycles of 94 °C 30 s, 55 °C 30 s, 72 °C 30 s; final 72 °C 5 min; held at 4 °C. Amplicons were sequenced by Tsingke Biotechnology (Beijing, China), and blasted for identity via NCBI (basic local alignment search tool—https://www.ncbi.nlm.nih.gov/BLAST (accessed on 29 February 2024)) and Ezbiocloud (https://www.ezbiocloud.net/identify (accessed on 29 February 2024)). Identified strains were stored at −80 °C in mGAM broth supplemented with 15% glycerol. Additional *Blautia* strains from the laboratory biobank, that were previously isolated from human feces [28], were also included in this study.

### 2.2. Screening for Cholesterol-Lowering Strains

Fresh bacterial culture (1% *v*/*v*) was inoculated into 200 µL mGAM broth with or without 100 µg/mL cholesterol (dissolved in anhydrous ethanol, Solarbio Science & Technology, Beijing, China) in a 96-well plate, and incubated at 37 °C for 24 h to investigate the effect of cholesterol on bacterial growth. The concentration of 100 µg/mL was selected in accordance with previous in vitro screening studies assessing microbial cholesterol removal [29,30]. OD_600_ was measured hourly using a SPECTROstar Omega microplate reader (BMG LabTech, Allmendgrün 8, 77799 Ortenberg, Germany). *Bacteroides thetaiotaomicron* HA2294 was included as a reference strain during the screening phase due to its established bile salt-modifying activity and reported ability to indirectly influence cholesterol availability through bile acid metabolism [31,32]. In this study, it served as a benchmark for cholesterol removal efficiency rather than as a model of direct cholesterol-to-coprostanol conversion.

The cholesterol-lowering ability was assessed for both live and heat-killed strains following established protocols [33,34] with modifications. Overnight bacterial pure culture (1% *v*/*v*) was inoculated into 5 mL mGAM broth containing 100 µg/mL cholesterol, while uninoculated broth served as a control. For heat-killed assays, autoclaved overnight bacterial culture was used after washing with PBS, pelleted (3720× *g*, 10 min), and resuspended to OD_600_ = 1 (~1 × 10^8^ CFU/mL). Autoclaved cells were also inoculated onto mGAM agar plates to verify complete inactivation. After incubation, supernatants were collected with centrifuge (2690× *g*, 10 min, 4 °C) for cholesterol quantification using the o-phthalaldehyde method [35]. Additionally, cholesterol-removing activity of live cells was analyzed in media supplemented with 0.3% oxgall (Shanghai Yuanye Biotechnology Co., Ltd., Shanghai China) to mimic the intestinal environment, where bile acid concentrations range from 0.2 to 2% [36]. A standard curve (0–200 µg/mL; Y = 0.008136 × X + 0.01294, R^2^ = 0.9932) was calculated. Cholesterol removal (%) was calculated as

$$Cholesterol\;removal\;(\%)=\frac{(C0-C1)\;}{C0}\times 100$$

where *C*0 and *C*1 are cholesterol concentrations in control and inoculated media, respectively.

### 2.3. Cholesterol Metabolism Analysis of Blautia

To prepare samples for GC-MS analysis, samples were prepared by inoculating 1% (*v*/*v*) of fresh overnight cultures into 2 mL mGAM broth, either supplemented with 100 µg/mL cholesterol or without cholesterol (control). Uninoculated mGAM broth containing 100 µg/mL cholesterol served as a media control. Cultures were incubated anaerobically at 37 °C for 24 h. After incubation, 2 mL ethanol and 2 mL 50% KOH (Sinopharm Chemical Reagent Co., Ltd., Shanghai, China) were added to the bacterial suspension, followed by sonication for 15 min in cycles of 4 s on and 4 s off. Samples were spiked with 10 µL 5α-cholestane (10 mg/mL, internal standard, Shanghai Macklin Biochemical Co., Ltd., Shanghai, China) and incubated at 60 °C for 1 h for saponification following established and previously validated protocols for cholesterol quantification in biological matrices [37,38], in which recovery and detection limits were previously evaluated. The internal standard was used to correct for potential variability during saponification, extraction, and injection. After cooling, 2 mL methyl tert-butyl ether (MtBE, Aladdin Scientific, Shanghai, China) was added to extract total lipids, and 1 mL of the organic phase was transferred to a tube and evaporated under a gentle stream of nitrogen gas. The resulting lipid pellets were re-dissolved in 1 mL MtBE. GC-MS analysis was performed as described previously on a Q Exactive GC Orbitrap GC-MS/MS system (Thermo Fisher Scientific, Waltham, MA, USA) [37,39]. One microliter of each sample was injected in splitless mode at 300 °C. Separation was achieved on a TG-5 SILMS semi-std non-polar column (30 m × 0.25 mm × 0.25 µm, Thermo Fisher Scientific, Waltham, MA, USA) with helium as the carrier gas (1.4 mL/min). Oven temperature was programmed as follows: 50 °C for 1 min; ramp to 180 °C at 25 °C/min and held for 2 min; ramp to 260 °C at 4° C/min and held for 15 min; ramp to 310 °C at 20 °C/min and held for 9.5 min (total runtime: 55 min). Mass spectrometry was conducted in electron ionization (70 eV) mode with a transfer line and ion source at 200 °C. Positive ion spectra were collected across m/z 34–500 at 60,000 resolution with a 3 min solvent delay. Metabolites were identified using automated spectral matching by xcalibur Qual browser and TraceFinder 5.1 General Quan (Thermo Fisher Scientific, Waltham, MA, USA) against NIST20 database, replicate, and GC-Orbitrap libraries with a mass accuracy threshold of <5 ppm. Metabolite distributions were visualized using ComplexHeatmap in R v4.4.2 [40,41,42]. Peak areas were log1p-transformed [ln(Area + 1)] to stabilize variance and optimize color scaling for heatmap visualization. These analyses provide descriptive profiling without inferential statistical testing or multiple correction across metabolites.

For LC-MS analysis, *Blautia hominis* HA2291 was cultured in mGAM broth with or without 100 µg/mL cholesterol, alongside a cholesterol-supplemented medium control. *Escherichia coli* BL21 (DE3) cells expressing recombinant RS03310, its mutants, or harboring the empty pET24a vector were grown in auto-induction medium (0.5% yeast extract, 25 mM Na_2_HPO_4_, 25 mM KH_2_PO_4_, 50 mM NH_4_Cl, 2 mM MgSO_4_, 0.05% glucose, 2% lactose, 0.5% glycerol; pH 7.2 [43]) supplemented with cholesterol for 24 h at 37 °C. Total lipids were extracted using MtBE as described above. Biological replicates were pooled prior to solvent evaporation. The combined extracts were dried under a gentle stream of nitrogen, re-dissolved with 1 mL methanol, and analyzed by Ultra-High Performance liquid chromatography system (SCIEX, ExionLC, UHPLC) coupled with a triple quadrupole mass spectrometer (SCIEX Triple Quad 5500+ QTrap Ready, AB Sciex Pte. Ltd., Singapore 739256, Singapore). Sterols were separated on a kinetex C18 100 Å column (2.1 × 100 mm, 2.6 µm) (Phenomenex, Tianjin, China) with water (A) and methanol (B) as mobile phases. After injecting 3 µL, the gradient was: 92% B, increased to 96% B over 7 min, held for 2 min, ramped to 100% B for 3 min, and then returned to 92% B for a 3 min re-equilibration. Flow rate was 0.3 mL/min and column temperature was 15 °C. The mass spectrometer was operated in positive APCI using the multiple reaction (MRM) mode with a dwell time of 50 ms per transition. The source/gas-dependent parameters were optimized and set as follows: curtain gas (CUR) 35 psi; collision gas (CAD) 9 psi; temperature (TEM) 350 °C; ion source gas 1 (GS1) 60 psi; ion source gas 2 (GS2) 60 psi; and Nebulizer Current (NC) 3 μA. The mass transitions and compound-dependent parameters were optimized using the authentic standards (Supplementary Table S1). Because biological replicates were pooled, LC-MS measurements represent composite samples and were interpreted qualitatively without inferential statistical analysis.

### 2.4. Transcriptome Analysis and Cholesterol-Lowering Related Gene Function Investigation

Fresh suspension (1% *v*/*v*) of *B. hominis* HA2291 (OD_600_ = 0.6) was inoculated into 100 mL mGAM broth with or without 100 µg/mL cholesterol and incubated anaerobically at 37 °C for 48 h to characterize gene expression profiles under sustained cholesterol exposure and metabolic adaptation conditions. Bacterial cells were harvested by centrifugation (5000× *g*, 10 min, 4 °C) and snap-frozen in liquid nitrogen. Total RNAs were extracted and sequenced by Novogene Co., Ltd. (Beijing, China) on Illumina Novaseq 6000 platform, San Diego, CA, USA.

The gene RS03310 (sterol-binding domain-containing protein-2) from *B. hominis* HA2291 was codon-optimized for *Escherichia coli* expression and cloned into a pET24a vector flanked by the EcoRI and XhoI restriction sites, synthesized by Tsingke Biotechnology (Beijing, China). The synthesized plasmid was transformed into *E. coli* BL21(DE3) and grown in LB broth with 50 µg/mL kanamycin antibiotics at 37 °C to OD_600_ = 0.5–0.6. Protein expression was induced with 0.15 mM IPTG (Isopropyl beta-D-thiogalactopyranoside) at 16 °C overnight. Recombinant His-tagged proteins were purified using IDA-Nickel magnetic beads (Solarbio Science & Technology, Beijing, China) according to the manufacturer’s instructions. The pooled elution fractions were dialyzed against refolding buffer (50 mM Tris-HCl, pH 8.0; 100 mM NaCl; 1 mM EDTA; 2 mM GSH; 0.2 mM GSSG). In parallel, an equivalent volume of refolding buffer (without protein) was placed in dialysis tubing and processed under identical conditions to generate a matched buffer control for downstream analyses. Refolded proteins and buffer controls were concentrated using Amicon Ultra centrifugal filters (10 kDa MWCO, Merck Chemical Technology (Shanghai) Co., Ltd., Beijing, China), quantified by absorbance at 280 nm, and verified by Western blotting.

Protein–cholesterol binding was assessed using a sedimentation-based assay modified from the liposome binding protocol described by Reginald and Chew [44]. In contrast to the original method, a total of 0.1 mg of RS03310 or bovine serum albumin (BSA) was added to 200 µL of suspension containing increasing amounts of cholesterol (5, 10, 20, or 40 µL of a 10 mg/mL cholesterol suspension) in 50 mM Tris-HCl buffer (pH 8.0). The mixture was incubated at 37 °C for 30 min and centrifuged (21,380× *g*, 30 min). Forty microliters of the supernatant were mixed with loading buffer. The pellet was washed, centrifuged (21,380× *g*, 5 min), resuspended in SDS-PAGE buffer, and heated (95 °C, 10 min). Ten microliters of each fraction were separated by 12.5% SDS-PAGE and stained to visualize bound and unbound protein. BSA served as a negative control.

Nicotinamide adenine dinucleotide (reduced, NADH) production was quantified spectrophotometrically at 340 nm according to the principle described by Kayamori et al. [45], which quantifies NADH using its molar extinction coefficient (ε = 6.22 × 10^3^ L·mol^−1^·cm^−1^). Reaction conditions were optimized for purified RS03310 as detailed below. 0.1 mg RS03310 was added to 200 µL suspension contained 50 mM Tris-HCl (pH 8.0), 100 µg/mL cholesterol, 4 mM NAD^+^, and 1 µg/mL BSA. Absorbance at 340 nm (A_340_) was measured at multiple time points (0–60 min). Four conditions were tested: (1) Control 1: buffer + BSA + NAD^+^ + cholesterol; (2) Control 2 (matrix blank): buffer + BSA + RS03310 storage buffer + NAD^+^ + cholesterol; (3) RS03310 (+NAD^+^): buffer + BSA + RS03310 + NAD^+^ + cholesterol; (4) RS03310 (–NAD^+^): buffer + BSA + RS03310 + cholesterol. A_340_ values were converted to NADH concentrations using a calibration curve (Y = 0.004038X + 0.4170; R^2^ = 0.9986). Specific activity was determined from the linear phase of product formation. To define the optimal regression window, multiple candidate time intervals (0–20, 0–30, 5–25, 15–45, 0–45, and 10–60 min) were evaluated by linear regression for each biological replicate. For each interval, the slope, coefficient of determination (R^2^), adjusted R^2^, and residual standard error (RSE) were calculated (Supplementary Table S2). Raw kinetic traces used for the regression analyses are provided in Supplementary Table S3. The 15–45 min interval was selected for activity determination based on maximal mean R^2^ across replicates (representative R^2^ = 0.9254), minimal residual variance, stable slope estimates across replicates, and absence of systematic residual curvature. This window captured the steady-state linear phase while excluding early lag and late deceleration phases. Specific activity was calculated as

$$\begin{array}{c} \mathrm{Specific}\;\mathrm{activity}\\ =\frac{\left({\mathrm{slope}}_{\left(\mathrm{RS}03310+\mathrm{NAD}\right)}-{\mathrm{slope}}_{\mathrm{Control}2}\right)(\mu g/mL/min)\times 1.507\;(\mu M/(\mu g/mL))\times 0.0002\;L)}{0.10\;(mg)} \end{array}$$

where slope is expressed in µg/mL/min, 1 µg/mL NADH = 1.507 µM, total protein = 0.10 mg, and total reaction volume = 200 µL (0.0002 L). The results are reported as µmol/min/mg (mean ± SD, *n* = 4 biological replicates, each the mean of 4 technical replicates). The limit of detection (LOD) for enzyme activity was determined from measured NADH calibration curve blank values. Using the slope of the blank signal (0.0291 µg/mL/min), the specific activity LOD was calculated as 0.000087 µmol/min/mg.

### 2.5. Statistical Analysis

All statistical analyses were performed using GraphPad Prism version 9.5.1 (GraphPad Software, San Diego, CA, USA) and R version 4.4.2. Standard curve equations were generated by linear regression analysis. Unless otherwise stated, data are presented as mean ± standard deviation (SD) from at least three independent biological replicates, each with technical replicates as specified in the figure legends. Statistical comparisons between two or more groups were performed using one- or two-way analysis of variance (ANOVA), followed by post hoc comparisons using Bonferroni’s or Dunnett’s multiple comparisons tests, as appropriate. Substrate-ligand interaction data were analyzed using a one-site specific binding model with nonlinear regression fitting. A significance threshold of α ≤ 0.05 was applied to all analyses.

## 3. Results

### 3.1. Evaluation and Screening of Blautia Strains That Are Efficient for Cholesterol Removal

**Figure 1:**
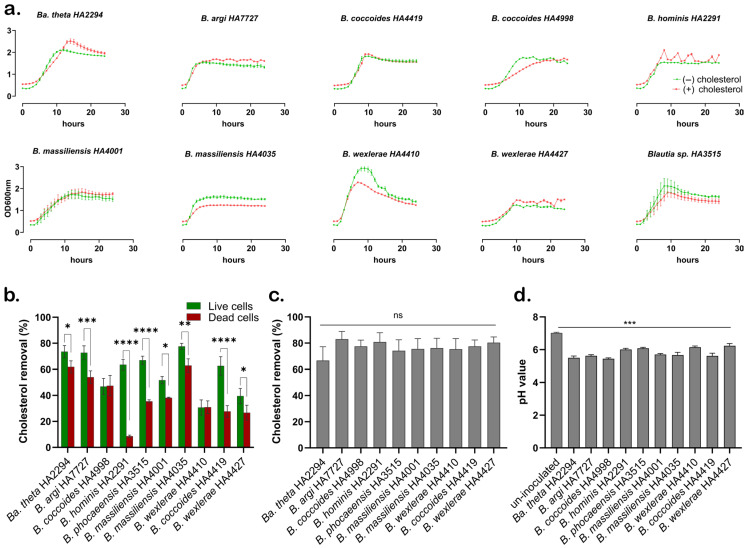
Characterization of cholesterol metabolism by *Blautia* strains. (**a**) Growth curves of strains cultured with (red) or without cholesterol (green). (**b**) Cholesterol removal by live versus heat-killed cells; the bars represent cholesterol removal percentage (%) of live cells (green) and dead cells (red) of bacterial strains. (**c**) Cholesterol removal by live cells with 0.3% oxgall. The bars represent cholesterol removal percentage (%), “ns” indicates no significance among all strains. (**d**) pH of spent culture medium from (**c**). The bars represent pH values of different strains. Data in panels (**b**–**d**) are presented as mean ± SD (*n* = 3 biological replicates) from two independent experiments. Statistical analyses were performed using two-way ANOVA followed by Šídák’s multiple comparisons test (panel (**b**)) and one-way ANOVA followed by Dunnett’s multiple comparisons test (panels (**c**,**d**)). Asterisks indicate *p* < 0.05 (*), *p* < 0.01 (**), *p* < 0.001 (***), *p* < 0.0001 (****).

A total of 63 *Blautia* strains obtained from newly isolated human fecal samples and from a laboratory biobank of previously isolated human gut strains [28] were identified according to their 16S rRNA gene sequences (Table S4) and were tested for their capacity for cholesterol metabolism. Growth curve analysis showed that supplementation with cholesterol did not inhibit the growth of any strain (Figure 1a). Among all tested 63 *Blautia* strains, live cells of nine strains removed approximately 31–78% of cholesterol from the medium, while the remaining 54 strains removed <5% (Table S4). To determine whether the strains’ cholesterol-lowering effect is due to active absorption rather than passive adsorption, we also tested the effect of dead cells on reducing cholesterol in the culture medium. The live and heat-killed cells of the reference strain *Bacteroides thetaiotaomicron* (*Ba. theta*) HA2294 removed 74% and 62% cholesterol, respectively (Figure 1b). For seven of the nine *Blautia* strains, cholesterol removal was significantly higher in live than in heat-killed cells [e.g., *B. hominis* HA2291 (64% vs. 8%), *Blautia* sp. HA3515 (67% vs. 34%), and *B. coccoides* HA4419 (63% vs. 28%)]. In contrast, *B. coccoides* HA4998 showed similar activities in live and heat-killed cells (46% vs. 47%, not significant). Similar strain-specific patterns were observed for *B. massiliensis* (HA4001 vs. HA4035) and *B. wexlerae* (HA4410 vs. HA4427), highlighting that cholesterol-removal capacity and underlying mechanisms are strongly strain-dependent. The potential contribution of cholesterol coprecipitation with bile acids was assessed. Under these conditions, cholesterol removal by the nine *Blautia* strains increased to 74–83% compared with 67% for *Ba. theta* HA2294; however, these differences did not reach statistical significance (Figure 1c). The final pH of culture supernatants ranged from 5.6 to 6.2 for most strains, with *B. coccoides* HA4998 reaching pH 5.4 while *Ba. theta* HA2294 reaching pH 5.5 (Figure 1d). Given that coprecipitation of cholesterol with deconjugated bile acids is reported to occur mainly at pH values below 5.5 [46,47], these data suggest that pH-driven precipitation contributed little to the observed cholesterol removal under our conditions. Taken together, these findings indicate that the *Blautia* strains exhibit strain-dependent cholesterol-removal activity, with *B. hominis* HA2291 emerging as a highly effective strain and therefore selected for subsequent mechanistic analyses.

### 3.2. Metabolomic Analysis of the B. hominis HA2291 Strain in the Presence of Cholesterol

**Figure 2:**
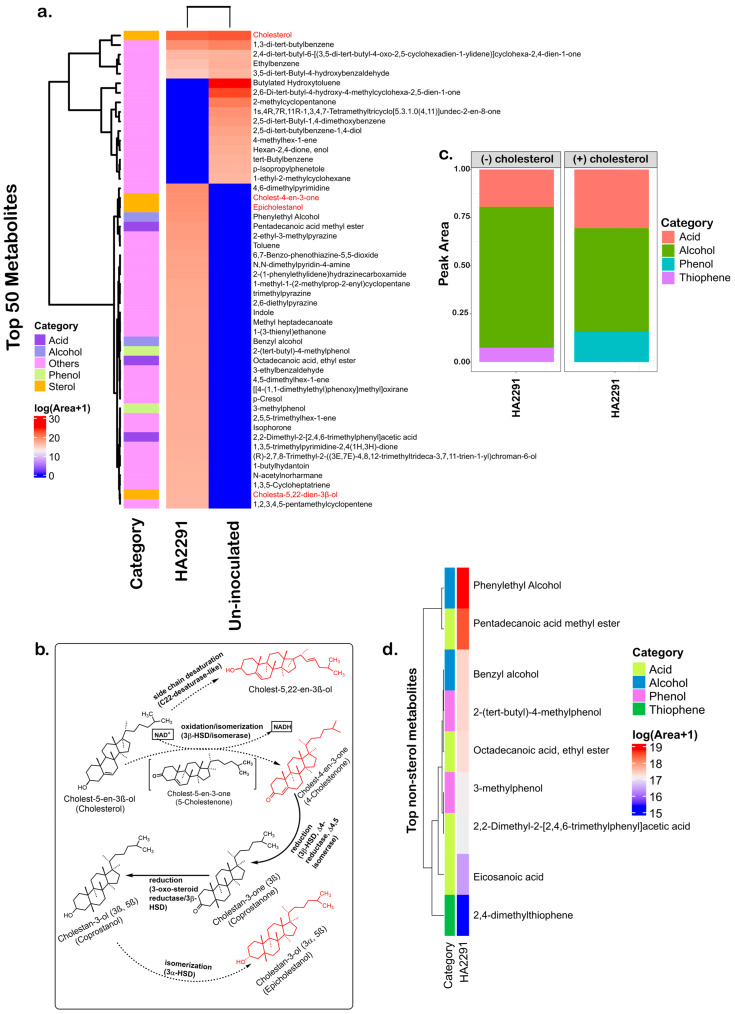
Absorption and degradation of cholesterol by the *B. hominis* HA2291 strain with GC-MS analysis. (**a**) Heatmap cluster of top 50 metabolites with different colors indicate different compounds: acid, alcohol, phenol, sterol and others with the color scale represents log1p-transformed peak areas (ln[Area + 1]) with values ranging from 0 (blue) to ~30 (red), reflecting the relative abundance of metabolites across samples. Sterol compounds are highlighted in red. (**b**) Proposed cholesterol transformation pathway into coprostanol by the HA2291 strain. Chemical structure derivatives in red were detected in GC-MS. Solid arrows indicate canonical intermediates in microbial cholesterol catabolism, whereas dotted arrows represent the proposed derivative routes specific to HA2291. 3β-HSD: 3β-hydroxysteroid dehydrogenase (**c**) Stacked bar plot showing non-sterol metabolites in the presence (+) or absence (−) of cholesterol, with colors indicating compound categories (acids, alcohols, phenols, and thiophenes) based on peak area. (**d**) Heatmap of the most abundant non-sterol metabolites, clustered by relative abundance. Colors represent log1p-transformed peak areas, ranging from 15 (blue) to 19 (red).

We next performed a metabolomic analysis of *B. hominis* HA2291 in the presence of cholesterol. We observed a reduced cholesterol-associated signal with the inoculation of HA2291 relative to un-inoculated controls (Figure 2a). Signals corresponding to several cholesterol-derived metabolites, including cholest-4-en-3-one, cholesta-5,22-diene-3β-ol, and epicholestanol, were detected exclusively in cholesterol-supplemented HA2291 culture (Figure 2a,b and Figure S1b,c, and Table S5), whereas coprostanone and coprostanol were not detected (Figure 2b). Moreover, cholesterol supplementation was associated with a marked remodeling of the global metabolic profile of HA2291, characterized by a shift from alcohol- to acid-dominated fermentation products (Figure 2c). Relative signal intensities of several saturated fatty acids, including pentadecanoic acid (C15:0), octadecanoic acid (C18:0), eicosanoic acid (C20:0), and the branched aromatic acid 2,2-dimethyl-2-[trimethylphenyl] acetic acid, were increased (Figure 2d). Phenolic compounds increased concurrently, while thiophene derivatives reduced to below the detection limit (Figure 2d).

### 3.3. Transcriptomic Analysis of Cholesterol Metabolism

**Figure 3:**
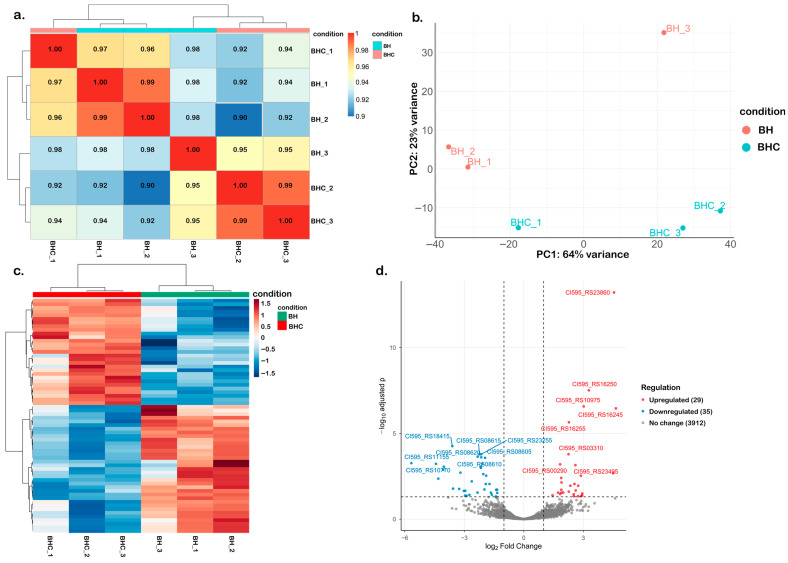
Exploratory analysis of transcriptomic analysis of *Blautia hominis* under BH and BHC conditions. (**a**) Heatmap of Pearson correlation coefficients (*r*) among biological replicates, with the color scale ranging from blue (*r* = 0.9) to red (*r* = 1.0). (**b**) Principal component analysis (PCA) separating BH and BHC samples. (**c**) Hierarchical clustering heatmap of differentially expressed genes (DEGs) across BH and BHC samples. The color scale represents row-wise Z-score-scaled gene expression values derived from log_2_ (FPKM + 1), with the color scale limited from blue (−1.5) to red (+1.5). (**d**) Volcano plot showing DEGs between BH and BHC samples. The abscissa represents log_2_ fold changes, and the ordinate represents statistical significance expressed as −log_10_ adjusted *p*-values. Red dots represent upregulated DEGs, blue dots represent downregulated DEGs, and gray dots represent non-significantly expressed genes. Dashed lines indicate the thresholds used to define differential expression (|log_2_FC| ≥ 1 and adjusted *p*-value < 0.05, shown as −log_10_(padj)).

**Figure 4:**
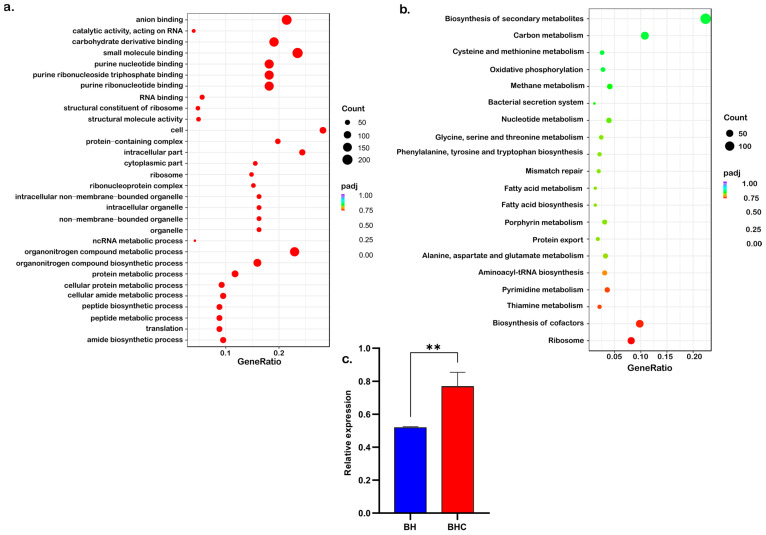
Functional enrichment and validation of transcriptomic changes in *Blautia hominis* under BH and BHC conditions. (**a**) Dot plot of the top 20 enriched Gene Ontology (GO) terms of DEGs. (**b**) Dot plot of the top 20 enriched KEGG pathways of DEGs. For panels (**a**,**b**), dot size corresponds to the number of genes, and different colors indicate adjusted *p*-values from 0.00 (red) to 1.00 (purple) with significance at *p* < 0.05. (**c**) The bars represent reverse transcription polymerase chain reaction (RT-PCR) analysis of RS03310 gene expression in the presence (BHC, red) or absence (BH, blue) of cholesterol. Data are presented as mean ± SD (*n* = 3 biological replicates) from two independent experiments. ** *p* < 0.01.

We analyzed transcriptomic profiles of HA2291 in cholesterol-enriched (BHC) and cholesterol-free (BH) media. RNA-seq analysis [48,49,50] generated libraries with an average of 7.45 million clean reads per sample, with Q20 and Q30 scores of 97.2% and 92.4%, respectively (Table S6). >85% alignments were mapped to the reference genome for both treatments, confirming sample purity, sequencing quality, and genome suitability (Table S7). Exploratory analysis [51,52] showed strong biological replicate consistency (Pearson *r* = 0.90–0.99; Figure 3a). PCA clearly separated BHC from BH samples (PC1 = 64% variance, PC2 = 23%), supported by hierarchical clustering (Figure 3b,c). Among 5116 annotated genes (Table S8), 29 were upregulated and 35 were downregulated in BHC samples (|log_2_FC| ≥ 1, adjusted *p* ≤ 0.05, Figure 3d). Notably, no significant enrichment for lipid metabolism genes was observed with neither GO (Figure 4a) nor KEGG pathway (Figure 4b) analyses. However, an upregulated gene (RS03310) potentially involved in sterol transport and conversion was identified (Figure 3d), whose expression was verified with qPCR (Figure 4c).

### 3.4. Cholesterol-Lowering Related Gene Functional Assessment

Phylogenetic analysis positioned RS03310 within monophyletic clade comprising *Blautia*-derived sterol carrier protein 2 (SCP2) homologs with high confidence (Shimodaira–Hasegawa approximate likelihood ratio test (SH-aLRT) = 99%), which resolved into three well-supported subclades (SH-aLRT values of 84%, 93%, and 89%, respectively; Figure S2a) [53]. Specifically, RS03310 clustered with *Blautia* orthologs WP_095171670 and WP_104804453 within the 89%-supported subclade. Notably, all *Blautia* SCP2 proteins were clearly separated from non-*Blautia* SCP2 homologs. Consistent with this finding, a sequence similarity network (SSN) analysis showed that RS03310-related sequences were widely distributed across diverse taxa but formed distinct clusters with *Blautia* sequences (Figure S2b) [54,55,56]. Furthermore, protein–protein interaction analysis predicted RS03310 association with RS03300, a condensation domain-containing protein, suggesting a potential functional partnership (Figure S2c).

**Figure 5:**
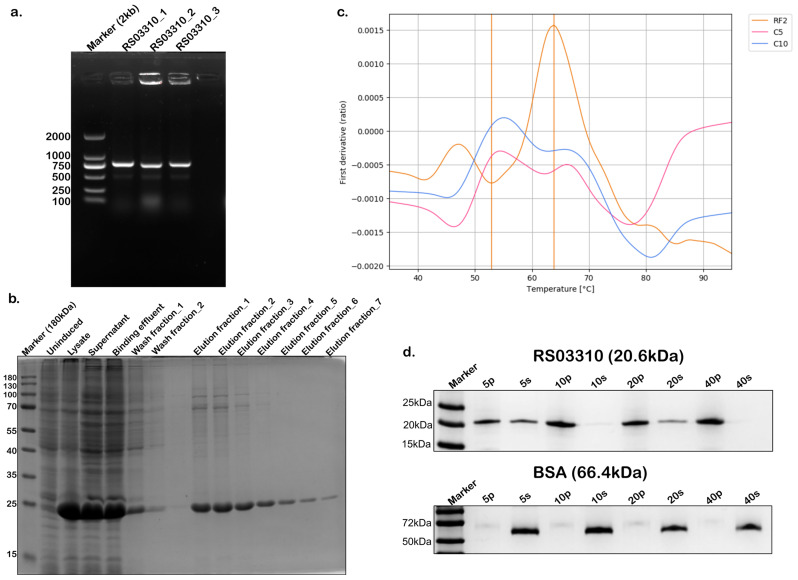
Gene cloning, protein expression and activity assessment of RS03310. (**a**) Agarose gel electrophoresis of RS03310 PCR amplicons from positive clones (expected size, 795 bp). (**b**) SDS-PAGE analysis of RS03310 protein expression and purification, showing bands at the expected molecular weight (~20.6 kDa). (**c**) Thermal unfolding monitoring showing two unfolding transitions at ~53 °C and ~64 °C of RS03310 in the absence of cholesterol (orange, RF2) and presence of cholesterol (red, C5; and blue, C10). Vertical lines in orange represent inflection temperature. (**d**) Cholesterol-binding activity assay of RS03310 (top) and bovine serum albumin (BSA; negative control, bottom); p (pellet) indicates protein fractions bound to cholesterol, and s (supernatant) indicates unbound protein fractions.

Successful cloning of the recombinant RS03310 gene was confirmed by agarose gel electrophoresis (Figure 5a). The expressed and purified protein (Figure 5b) was refolded by buffer exchange (see Methods). Thermal unfolding of the refolded protein was monitored by measuring the intrinsic fluorescence of tyrosine and tryptophan residues [57]. In the absence of cholesterol, the unfolding profile exhibited two distinct transitions at ~53 °C and ~64 °C. Strikingly, these transitions were abolished upon incubation with cholesterol, indicating a stabilization of the protein structure (Figure 5c). Furthermore, a pellet-based binding assay demonstrated a concentration-dependent recruitment of RS03310 to the cholesterol-containing pellet fraction, confirming the specific interaction between RS03310 and cholesterol (Figure 5d).

### 3.5. Site-Directed Mutagenesis for RS03310 and Functional Analysis

In silico modeling revealed that the 3D structure of RS03310 shared 63% identity with the rabbit SCP2 template (PDB ID: 1C44) [58,59,60], featuring a five-stranded antiparallel β-sheet flanked by five α-helices (Supplementary Figure S3a). Blind docking predicted cholesterol binding with free energy of −9.16 kcal/mol (AutoDock 4.2.6) [61,62,63] and −9.3 kcal/mol (CB-Dock2) [64,65,66], stabilized by a hydrogen bonding (GLY21) and hydrophobic interaction (π–σ, alkyl, and π–alkyl) within a binding pocket involving residues ILE10, ILE22, TYR28, PHE30, LEU101, ASN103, PHE104, GLN107, ALA108, and ASN109 [67]. In silico site-directed mutagenesis (F104S, Q107K, A108S, N109K, G21C and I22T), and *C*-terminal truncation within the SCP2 motif, despite inducing a structural reorganization into an ααβββ fold (Supplementary Figure S3b–d), preserved cholesterol binding via alternative mechanisms, such as a π–σ interaction with PHE63 (3.4 Å) or interactions with other residues.

**Figure 6:**
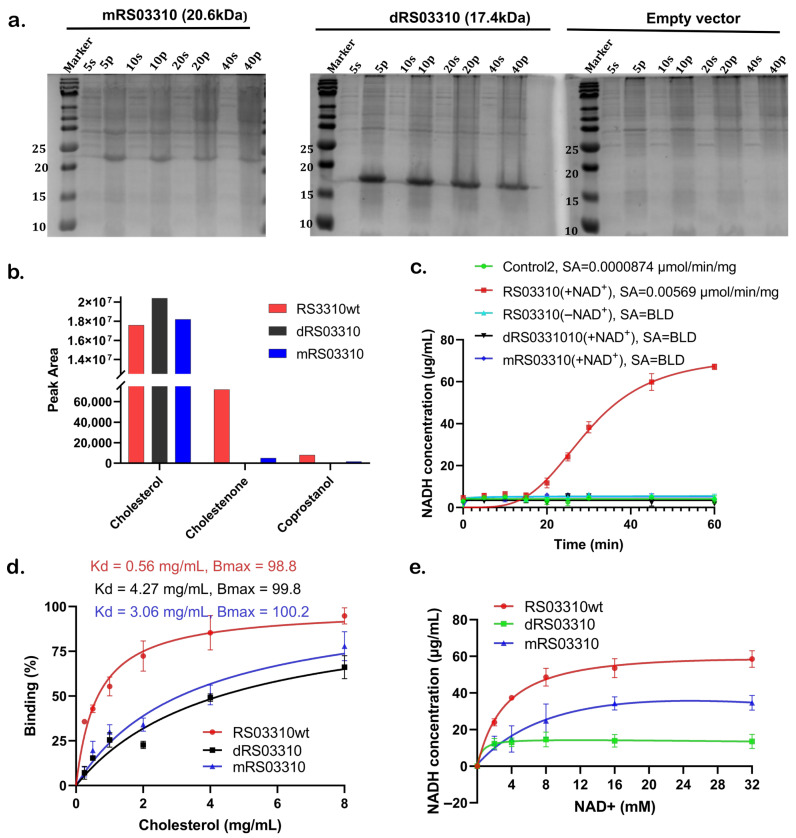
Functional assessment of RS03310wt and its variants. (**a**) Cholesterol-binding assay in crude cell lysates. SDS-PAGE of mRS03310 (left), dRS03310 (middle) and *E. coli* BL21 (DE3) cells with empty vector (negative control, right); p (pellet) indicates protein fractions bound to cholesterol, and s (supernatant) indicates unbound protein fractions. (**b**) Targeted LC-MS analysis of total sterol extracts from cells expressing RS03310 wild-type (wt; red), dRS03310 (black), mRS03310 (blue), and normalized to the empty vector control. (**c**) Enzymatic activity of RS03310 and mutant proteins in the presence and absence of NAD^+^, measured by NADH production. Linear regression was performed within the empirically defined linear phase (15–45 min). Slopes derived from the linear window were used to calculate specific activity (SA) and establish the assay limit of detection, as detailed in Section 2 and Table S8. Data are presented as mean ± SD (*n* = 4). BLD: below limit of detection. (**d**) Saturation binding assay performed with increasing cholesterol concentrations (0–8 mg/mL). (**e**) Endpoint NADH production by RS03310wt and its variants at increasing NAD^+^ concentrations. For panels (**c**–**e**), data points represent mean ± SD (*n* = 3), and nonlinear regression fits are shown as solid lines.

To validate these predictions, we constructed mutant recombinant genes through amino acid substitution (mRS03310) and truncation (dRS03310) of the predicted cholesterol-binding pocket (Supplementary Figure S4a–f, Table S9). Compared to empty vector controls, crude extracts from BL21 (DE3) transformants expressing these mutants retained cholesterol-binding activity (Figure 6a). LC-MS analysis of pooled cholesterol-supplemented cultures detected residual cholesterol in mutant transformants than in the wild-type. Signal intensity appeared higher in the mutants, consistent with reduced cholesterol turnover. Trace sterol intermediates were observed in wild-type and substitution mutant (Figure 6b). These LC-MS data are qualitative in nature. Enzymatic assays using purified proteins demonstrated that only RS03310wt produced NADH in a time-dependent manner, exhibiting a specific activity of 0.00569 ± 0.002 µmol/min/mg (5.69 nmol/min/mg). The empirically determined activity detection threshold, derived from Control2 reactions, was 0.0000874 ± 0.0018 µmol/min/mg (0.0874 nmol/min/mg), yielding a signal-to-background ratio of approximately 65-fold. No detectable NADH production was observed in the absence of NAD^+^ or in mutant proteins. The corresponding slopes for RS03310 (-NAD^+^) and mutant variants (0.013–0.019 µg/mL/min) were below the empirically defined slope detection threshold (0.0291 ± 0.00013 µg/mL/min), indicating that their activities were indistinguishable from background under these assay conditions (Figure 6c, Supplementary Figure S5a). Saturation binding experiments indicated high-affinity cholesterol binding for RS03310wt (Kd ~0.56 mg/mL) compared with markedly lower affinities for mutants (Kd ~3.06 and 4.27 mg/mL), while Bmax values remained similar (Bmax ~98–100%, Figure 6d). Endpoint activity assays revealed a near-linear increase in NADH production by RS03310wt between 2-16 mM NAD^+^ followed by a plateau up to 32 mM NAD^+^, whereas both mutants responded minimally over the same NAD^+^ concentration range (Figure 6e). Together, these data suggest that RS03310 is an SCP2-like protein that binds cholesterol and exhibits NAD^+^-dependent redox activity in the presence of cholesterol, in contrast to canonical SCP2 proteins, which function primarily as lipid carriers and are not known to require NAD^+^ [68,69,70,71].

## 4. Discussion

Growing recognition that gut microbes modulate host lipid metabolism provided the conceptual foundation for this study. Previous work linked *Blautia* abundance to improvements in obesity, diabetes, and visceral fat accumulation [22,72,73,74], suggesting a potential role in cholesterol regulation. However, other reports have implicated *Blautia* as a risk genus for obesity [20], indicating that probiotic potential may be strain-specific, and information on the cholesterol-reduction potential of *Blautia* remains limited. In this study, we assessed cholesterol removal by different *Blautia* strains isolated from human feces using an in vitro approach. We observed cholesterol removal by *Blautia* strains through both cell-surface adsorption (demonstrated by 8–63% removal by heat-killed cells) and additional live cell-dependent processes (total removal 31–78%), suggesting metabolic activity contributes substantially beyond passive binding. Given the final culture pH of 5.6–6.2, the enhanced cholesterol removal (74–83%) with 0.3% oxgall supplementation is likely due to bile-mediated interactions, rather than cholesterol coprecipitation [43,47].

Furthermore, the levels of cholesterol removal were highly variable among strains of the same species, supporting the notion that cholesterol reduction is a strain-specific characteristic. In this study, *B. hominis* HA2291 cells demonstrated a significant capacity for cholesterol absorption, and metabolomic profiling detected cholest-4-en-3-one and epicholestanol, which are normally known as intermediate products of cholesterol metabolism. Cholest-4-en-3-one is a recognized early intermediate sterol formed by 3β-hydroxysteroid dehydrogenase/isomerase or cholesterol oxidase [9,75], while epicholestanol is a C3-epimer (3α-hydroxy isomer) of coprostanol, is commonly generated through reductive conversion of coprostanone and subsequent epimerization steps during microbial cholesterol metabolism [76]. The presence of these compounds suggests that HA2291 employs an indirect cholesterol modification strategy analogous to that reported for *Eubacterium coprostanoligenes*, *Bacteroides dorei*, *Eubacterium* sp. and certain *Clostridium* cluster IV species [76]. We also detected cholesta-5,22-dien-3β-ol, a non-canonical derivative in microbial cholesterol biotransformation pathways, suggesting possible involvement of Δ22-desaturase-like activity [77], highlighting metabolic flexibility beyond known cholesterol-reducing pathways. Additionally, cholesterol exposure was associated with increased relative abundances of several saturated fatty acids, including pentadecanoic acid (C15:0), octadecanoic acid (C18:0, stearic acid), eicosanoic acid (C20:0, arachidic acid), and 2,2-dimethyl-2-[trimethylphenyl]acetic acid. These shifts align with previous reports linking sterol utilization to alterations in lipid saturation and membrane composition [12] and support an association between cholesterol availability and broad reprogramming of cellular metabolism in the strain HA2291.

The transcriptome of HA2291 cells grown in cholesterol environment did not show any upregulation of canonical genes related to lipid catabolism. This possibly due to incomplete annotation of the reference genome or a true absence of a canonical lipid-catabolic response under the tested conditions. Previous studies have indicated that cholesterol oxidase and hydroxysteroid dehydrogenase are dispensable for cholesterol degradation [12,78]. The lack of canonical gene enrichment in lipid metabolism in this study may suggest alternative pathways for cholesterol degradation or that cholesterol does not trigger a catabolic response in HA2291, aside from conversion to coprostanol, as previously observed in *Bifidobacterium bifidum* PRL2010 [12]. Despite the lack of lipid metabolism gene upregulation, RS03310, a SCP-2-like protein, was detected upregulated in cholesterol-rich conditions. Moreover, biochemical assays confirmed that it can bind cholesterol directly and, catalyze its NAD^+^-dependent oxidation to cholest-4-en-3-one. Phylogenetic analyses place RS03310 firmly within a *Blautia*-specific SCP2 clade, underscoring its potential role in sterol transport or metabolism. The predicted interaction with the nearby condensation domain protein (RS03300) suggests a coordinated role in a multi-enzyme sterol modification pathway. Although, SCP-2 proteins are traditionally regarded as non-enzymatic lipid carriers [70,71,79], structural divergence and clustering analyses indicated that *Blautia* homologs have functionally diverged. Assessment of enzymatic kinetics using an optimized linear regression window (15–45 min) substantially improved precision and signal discrimination. The calculated specific activity (5.7 nmol/min/mg) was ~65-fold above the empirically defined activity detection threshold (0.087 nmol/min/mg), with strong linearity (R^2^ = 0.921 across replicates) and minimal residual variance. These parameters indicate that NADH production is robustly distinguishable from background and not attributable to analytical noise. Although the catalytic rate is modest compared with industrial cholesterol oxidases, it falls within the range reported for physiological bacterial cholesterol dehydrogenases [80], supporting its biological plausibility in a commensal metabolic context. Thus, these data support RS03310 as a cholesterol-interacting enzyme that contributes to cholesterol biotransformation in *Blautia hominis* HA2291, rather than a high-throughput catabolic enzyme. These findings expand the known metabolic capabilities of *Blautia* species and the functional repertoire of SCP-2 proteins. They indicate that *B. hominis* can sequester cholesterol from the environment and transforms it into intermediate metabolites. This metabolic versatility likely represents an adaptive advantage within the competitive gut ecosystem and may have implications for host lipid homeostasis.

Notwithstanding these mechanistic insights, several limitations of the present study should be acknowledged. First, all conclusions are drawn from in vitro experiments and may not fully capture host–microbiota dynamics in vivo. Validation in hyperlipidemic animal models, particularly using gene-knockout systems, will be necessary to establish the physiological relevance of the identified pathways. Second, although additional strains were obtained from a multi-donor biobank, the strain isolation was performed from a limited number of healthy donors. Consequently, the donor pool may not fully represent the breadth of interindividual microbiota diversity. Third, heat inactivation by autoclaving may alter bacterial surface architecture and potentially underestimate passive cholesterol adsorption compared to milder inactivation methods. However, this approach ensured complete metabolic inactivation and allowed separation of adsorption from active metabolism. Fourth, transcriptomic profiling was performed at a single 48 h time point following cholesterol exposure to capture sustained transcriptional adaptation. Early transient responses occurring at earlier time points (e.g., 24 h) were not assessed and may reveal additional cholesterol-responsive genes. Finally, LC-MS analyses were conducted on pooled samples; therefore, these measurements provide qualitative support for RS03310-dependent cholesterol biotransformation but do not permit inferential statistical comparison between samples.

## 5. Conclusions

In this study, we demonstrate that *Blautia hominis* HA2291 exhibits multiple in vitro mechanisms for cholesterol handling, including adsorption, bile-enhanced removal, and enzymatic biotransformation mediated in part by RS03310. These findings provide mechanistic insight into sterol processing by a commensal *Blautia* strain under controlled experimental conditions. Further in vivo studies across multiple strains and model systems will be required to determine whether these in vitro effects translate into improvements in serum lipid parameters, hepatic lipid homeostasis, and atherosclerotic lesion burden in established hyperlipidemic animal models.

## Data Availability

All data generated or analyzed from this study are included in this published article. RNA-seq raw and processed sequencing data generated in this study have been submitted to the NCBI Gene Expression Omnibus (GEO; https://www.ncbi.nlm.nih.gov/geo/) under accession number GSE316063.

## Supplementary Materials

The following supporting information can be downloaded at https://www.mdpi.com/article/10.3390/nu18060882/s1. Supporting material containing Figure S1. GC-MS and LC-MS chromatograms showing cholestenone production by *Blautia hominis* HA2291 grown with cholesterol; Figure S2. In silico analysis positioning RS03310 as a sterol carrier protein 2 (SCP2) domain-containing protein in *B. hominis* HA2291; Figure S3. Cholesterol docking and motif analysis of RS03310; Figure S4. Vector-based site-directed mutagenesis of RS03310 to generate a double mutant and a partial deletion; Figure S5. Linear regression-based time-window selection for enzymatic kinetic analysis; Table S1. The LC mass transitions; Table S2. Linear regression-based time-window selection for enzymatic kinetic analysis; Table S3. Raw traces for enzymatic kinetic analysis; Table S4. 16S rRNA sequences of *Blautia* strains and cholesterol removal screening results; Table S5. GC-MS output data for *B. hominis* HA2291; Table S6. Summary of sample sequencing data quality for BH (non-cholesterol environment) and BHC (in cholesterol environment); Table S7. Statistics on the alignment of samples with the reference genome of *Blautia producta* (ncbi_blautia_gcf_002270465_1_asm227046v1); Table S8: DEG analysis output for *B. hominis* HA2291; Table S9. Primers for vector-based site directed mutagenesis for pET24-RS03310.

## Abbreviations

The following abbreviations are used in this manuscript:

ANOVA	Analysis of variance
APCI	Atmospheric pressure chemical ionization
BH	*Blautia hominis* without cholesterol
BHC	*Blautia hominis* with cholesterol
BhSCP2	*Blautia hominis* sterol carrier protein 2
BSA	Bovine serum albumin
BSH	Bile salt hydrolase
C10	10 µL cholesterol
C5	5 µL cholesterol
CAD	Collision gas
CFU	Colony forming unit
CUR	Curtain gas
CVD	Cardiovascular disease
DEG	Differentially expressed genes
dRS03310	Truncated RS03310 protein
ev	Electron ionization
GC-MS	Gas chromatography mass spectrophotometry
GS1	Ion source gas 1
GS2	Ion source gas 2
IPTG	Isopropyl beta-D-thiogalactopyranoside
KOH	Potassium hydroxide
LC-MS	Liquid chromatography–mass spectrophotometry
mGAM	modified Gifu Anaerobic Medium
mRS03310	Mutated RS03310 protein by substitution
MtBE	methyl tert-butyl ether
NAD^+^	Nicotinamide adenine dinucleotide (oxidized)
NADH	Nicotinamide adenine dinucleotide (reduced)
NC	Nebulizer Current
OD	Optical density
PBS	Phosphate-buffered saline
PCA	Principal component analysis
qRT-PCR	Quantitative real-time polymerase chain reaction
SCFA	Short-chain fatty acid
SCP2	Sterol carrier protein 2
SD	Standard deviation
SDS-PAGE	Sodium Dodecyl Sulfate Polyacrylamide Gel Electrophoresis
SH-aLRT	Shimodaira–Hasegawa approximate likelihood ratio test
SSNs	Sequence similarity networks
TEM	Temperature
