# Supplementary material for: An In Vitro Functional Characterization of the Cholesterol-Transforming Blautia hominis Strain HA2291 Isolated from the Human Gut
Source: Nutrients. 2026 Mar 10;18(6):882. doi: 10.3390/nu18060882 (PMC13029680; doi:10.3390/nu18060882)
Supplement: Supplementary file 1 [file nutrients-18-00882-s001.zip › Supporting Material.pdf]

An in vitro functional characterization of the cholesterol-transforming *Blautia hominis* HA2291 isolated from the human gut

Warren Chanda [1,2], He Jiang [1,] * and Shuang-Jiang Liu [1,3,4,] *

[1]State Key Laboratory of Microbial Technology, Shandong University, Qingdao 266237, China; d2021077@mail.sdu.edu.cn

[2]Pathology and Microbiology Department, School of Medicine and Health Sciences, Mulungushi University, P.O Box 60009 Livingstone, Zambia

[3]State Key Laboratory of Microbial Resources, and Environmental Microbiology Research Center (EMRC), Institute of Microbiology, Chinese Academy of Sciences, Beijing 100101, China

[4]University of Chinese Academy of Sciences, Beijing 100049, China

*Correspondence: jianghe@sdu.edu.cn (H.J.); liusj@sdu.edu.cn (S.-J.L.)

Experimental section

RNA-seq analysis and Bioinformatics. Total RNA was extracted and assessed for integrity and purity. RNA degradation and contamination were evaluated on 1 % agarose gels, and RNA integrity was determined using the RNA Nano 6000 Assay Kit on the Bioanalyzer 2100 system (Agilent Technologies, USA). For library preparation, mRNA was purified from total RNA using oligo-dT probes to remove rRNA. Fragmentation was performed with divalent cations under elevated temperature in First Strand Synthesis Reaction Buffer (5×). First-strand cDNA was synthesized using random hexamer primers and M-MuLV Reverse Transcriptase, followed by RNase H digestion to remove RNA. Second-strand cDNA synthesis was performed using DNA polymerase I with dUTP substituted for dTTP. Overhangs were converted to blunt ends by exonuclease/polymerase treatment, followed by 3′-end adenylation and ligation of adaptors containing hairpin loop structures. USER enzyme treatment was applied to degrade the uracil-containing second strand. cDNA fragments of approximately 370-420 bp were size-selected using the AMPure XP system (Beckman Coulter, USA). The resulting libraries were amplified using Phusion High-Fidelity DNA polymerase with universal and index primers, purified again with AMPure XP, and assessed for quality on the Agilent Bioanalyzer 2100 system. Cluster generation was performed on a cBot Cluster Generation System using the TruSeq PE Cluster Kit v3-cBot-HS (Illumina) following the manufacturer's protocol. Libraries were sequenced on an Illumina NovaSeq platform to generate 150 bp paired-end reads.

Bioinformatics analysis. Raw sequencing reads in FASTQ format were quality-checked and filtered to remove adapter sequences, reads containing poly-N regions, and low-quality reads. Clean reads were evaluated for quality metrics (Q20, Q30, and GC content) and used for downstream analyses. Reads were aligned to the *Blautia producta* reference genome (*Blautia producta* NCBI accession: GCF_002270465.1_ASM227046v1) using Bowtie2 v2.2.3 [1]. Gene-level read counts were obtained with HTSeq v0.6.1 [2], and gene expression levels were normalized as fragments per kilobase of

transcript per million mapped reads (FPKM), accounting for gene length and sequencing depth to minimize technical bias [3].

Differential expression analysis was conducted using the DESeq2 R package (v3.0.3) [4]. *P*-values were adjusted for multiple testing using the Benjamini–Hochberg false discovery rate (FDR) method [5], and genes with adjusted $p < 0.05$ were considered differentially expressed. Functional enrichment analysis of differentially expressed genes was performed with GOseq (for Gene Ontology) after correction for gene-length bias, and with KOBAS (for KEGG pathway analysis). GO and KEGG terms with corrected $p < 0.05$ were considered significantly enriched. Gene set enrichment analysis (GSEA) was carried out using the GSEA software (Broad Institute, http://www.broadinstitute.org/gsea) with GO and KEGG datasets analyzed independently. Finally, protein-protein interaction (PPI) networks for the differentially expressed genes were constructed using the STRING database to explore predicted and known molecular interactions.

qRT-PCR validation of cholesterol-related genes. To validate RNA-seq results, qRT-PCR was performed for selected cholesterol-related gene that overexpressed under cholesterol-supplemented conditions. Reverse transcription was carried out using the HiScript III RT SuperMix (+gDNA wiper) kit (Vazyme, China) according to the manufacturer's instructions. One microliter of cDNA (equivalent to 50 ng RNA) was used per qRT-PCR reaction, performed with ChamQ Universal SYBR qPCR Master Mix (Vazyme, China) on a LightCycler® 96 (Roche). Gene-specific primers (Table S9) were designed with NCBI Primer-BLAST, synthesized by Tsingke Biotechnology (Beijing, China), and validated for specificity and amplification efficiency. The cycling conditions were: 95 °C for 30 s; 40 cycles of 95 °C for 10 s, 63 °C for 20 s, and 72 °C for 10 s. No-template controls (NTCs) and no-reverse-transcription controls (NoRTs) were included. Expression levels were normalized to the 16S rRNA housekeeping gene, confirmed to be stable under the experimental conditions.

In silico* analysis.* Sequence similarity network (SSN) for RS03310 were generated using the EFI-EST tool (https://efi.igb.illinois.edu/efi-est/) [6,7] with 100 % sequence identity and visualized in Cytoscape v3.10.2 [8]. Protein sequences from the UniProt database were analyzed with the Enzyme Function Initiative-Enzyme Similarity Tool (EFI-EST), using pairwise identity thresholds of 20% [6,7]. Networks of representative nodes (100 % sequence identity) were visualized in Cytoscape v3.10.2 [8]. SSN analysis allows for visualization of relationships among protein sequences and group related proteins based on sequence similarity, facilitating functional inference.

For phylogeny analysis, RS03310 protein sequence was blasted on BLAST/FASTA sequence similarity search against nr-aa (GenBank, UniProt, RefSeq and PDBSTR) database (https://www.genome.jp/tools/blast/; accessed on 2 December 2025). A phylogenetic tree was constructed using the Phylogeny.fr online server (http://phylogeny.lirmm.fr/phylo_cgi/index.cgi; accessed on 2 December 2025) [9] following this pipeline: the top 20 protein sequences showing highest similarity to RS03310 were aligned with MUSCLE (v3.7) using default parameters, and ambiguous regions (i.e., those containing gaps and/or poorly aligned residues) were removed with Gblocks (v0.91b). The phylogenetic tree was inferred using the maximum likelihood method implemented in PhyML (v3.0). The WAG substitution model was applied, assuming an estimated proportion of invariant sites and four gamma-distributed rate categories to account for site-specific rate heterogeneity. The gamma shape parameter was estimated directly from the data, and the reliability of internal branches was assessed using the aLRT (SH-like) test. Tree rendering was carried out with TreeDyn (v198.3), and final visualization was performed using MEGA12 software.

Protein-protein interaction (PPI) analysis was performed using the NovoMagic cloud platform (https://magic.novogene.com; accessed 14 October 2024) and visualized in Cytoscape.v3.10.2.

Western blotting. Proteins were separated on 12.5% SDS-PAGE gels and transferred to methanol-activated PVDF membranes (Beyotime Biotech, China) using transfer buffer (25 mM Tris base, 190 mM glycine, 0.04% SDS, 20% methanol) for 60 min at 300 mA on ice. Membranes were washed with distilled water for 5 min, blocked with QuickBlock solution (Beyotime) for 15 min at room temperature, and washed three times with TBST (Tris-buffered saline + 0.1% Tween-20). Membranes were incubated overnight at 4 °C with mouse anti-His-tag primary antibody (Beyotime), washed, and incubated for 60 min at room temperature with HRP-conjugated goat anti-mouse IgG secondary antibody.

Tycho NT.06 protein stability assay. Purified RS03310 was refolded by buffer exchange and its thermal stability, with or without cholesterol, was examined using the Tycho NT.06 system (NanoTemper) [10]. Samples were loaded into capillaries, and intrinsic fluorescence ratios (330 nm/350 nm) were measured as temperature increased from 35 °C to 95 °C at 30 °C/min. Inference temperature (Ti) values were calculated by the instrument software.

NADH calibration (A340). NADH disodium solutions (0-400 µg/mL) were prepared in 50 mM Tris-HCl (pH 8.0) containing 1 µg/mL BSA and 4 µL of RS03310 storage buffer per 200 µL reaction. Absorbance at 340 nm (A_{340}) was measured after 30 min at 37 °C in a Varioskan LUX plate reader (Thermo Scientific). Matrix blanks were subtracted, and calibration curves were generated by linear regression in GraphPad Prism 9.

Structural modeling and site-directed mutagenesis. No significant structural homology was identified for RS03310 in the RCSB Protein Data Bank (PDB). A 3D structure was predicted using the PHYRE2 server (Protein Homology/analogY Recognition Engine [11], with 99.33 % confidence and 63 % coverage relative to rabbit SCP-2 (PDB: 1C44) [12]). Ligand docking preparation, including hydrogenation, charge assignment, and ligand (.mol2) generation, was performed using AutoDockTools, UCSF Chimera 1.9, and Avogadro 1.2.0 [13-15]. Both RS03310.pdb and cholesterol.mol2 files were imported into Autodock4.2.6 software. Docking simulations were run in AutoDock 4.2.6 (100 LGA runs; 25 million evaluations) and validated using the CB-Dock2 server (https://cadd.labshare.cn/cb-dock2/index.php) that performs auto blind docking with autodock vina [16-18]. BIOVIA Discovery Studio 2025 Client was used to visualize complex model.

Active site mutagenesis and deletion constructs. Based on docking predictions, cholesterol-interacting residues were substituted with hydrophilic amino acids to reduce binding affinity. Conserved regions were predicted using PHYRE2 [19] and PI-Site, and a C-terminal region enriched with active-site and interface residues was selected for partial deletion. This approach targeted evolutionarily conserved domains likely to be essential for protein function. To probe the structural determinants of cholesterol binding, in silico site-directed mutagenesis was performed. Substitutions of F104S, Q107K, A108S, and N109K, along with G21C and I22T), were introduced (collectively 104FSKQAN109 → 104SSKKSK109 and 21GI22 → 21CT22) to alter pocket hydrophobicity and hydrogen bonding

Mutant plasmid construction. Like in-silico mutations, primers to introduce base substitutions of F104S, Q107K, A108S, N109K, G21C and I22T, and for truncating the protein (Table S7) were

designed with NCBI Primer-BLAST and synthesized by Tsingke Biotechnology (Beijing, China). The pET24-RS03310 plasmid was extracted using the TIANprep Mini Plasmid Kit (TIANGEN Biotech, Beijing). PCR reactions (40 µL) contained 50 ng plasmid DNA, 1 µL each primer (10 µM), and 20 µL 2× Phanta UniFi Master Mix (Vanzyme). Amplification was performed with an initial denaturation at 95 °C for 5 min; 32 cycles of 95 °C for 30 s, 58 °C for 30 s, and 72 °C for 90 s; and a final extension at 72 °C for 3 min. Amplicons were purified, ligated using Exnase II (ClonExpress II One Step Kit, Vanzyme), and transformed into *E. coli* DH5α cells by heat shock (42 °C, 90 s). Transformants were recovered in SOC medium (TransGen Biotech, China), plated on LB agar with 50 µg/mL kanamycin, and incubated overnight. Colony PCR screening and sequencing (Tsingke Biotechnology, Beijing) confirmed successful mutagenesis. Then, plasmid was extracted and transformed into *E. coli* BL21 (DE3). Protein expression was performed as described in the main paper. Crude lysates were normalized to cell pellet wet weight for downstream assays.

For saturation binding assays, a pulldown-based binding approach was used in which protein recovered in the pellet following cholesterol incubation was operationally defined as the bound fraction. Purified RS03310, dRS03310, or mRS03310 (1 mg/mL; 200 µL per reaction, 0.2 mg total protein) was incubated with increasing concentrations of cholesterol (0-8 mg/mL in 50 mM Tris-HCl, pH 8.0) at 37 °C for 30 min. Samples were centrifuged at 21,380 x g for 30 min, and pellets were washed twice with PBS (pH 8.0) to remove unbound cholesterol, followed by a second centrifugation at 15,000 ×g for 5 min. Pellets were resuspended in SDS-PAGE loading buffer and heated at 95 °C for 10 min. Ten microliters of each sample were resolved by 12.5 % SDS-PAGE and stained to visualize recovered protein. A no-cholesterol control processed under identical conditions was included in each experiment. Band intensities were quantified using ImageJ. Binding was expressed as mean ± SD (n = 3) and normalized to the no-cholesterol control according to:

$$Binding\ (\%) = \frac{mean\ band\ intensity\ (cholesterol - treated)}{mean\ band\ instensity\ (no - cholesterol\ control)} X100$$

The apparent dissociation constant (Kd) was estimated by fitting a one-site specific binding (saturation) model in GraphPad Prism 9.

For the endpoint NADH production assay, 0.1 mg of purified RS03310, dRS03310, or mRS03310 was added to 200 µL of reaction mixture containing 50 mM Tris-HCl (pH 8.0), 100 µg/mL cholesterol, and 1 µg/mL BSA, with NAD^+ varied from 0 to 32 mM. Reactions were incubated at 37 °C for 30 min, and absorbance at 340 nm was measured to quantify NADH formation. NADH concentrations were calculated from a standard curve (R^2 = 0.9837, p < 0.001). Data are presented as NADH production (mean ± SD, n = 3 independent experiments).

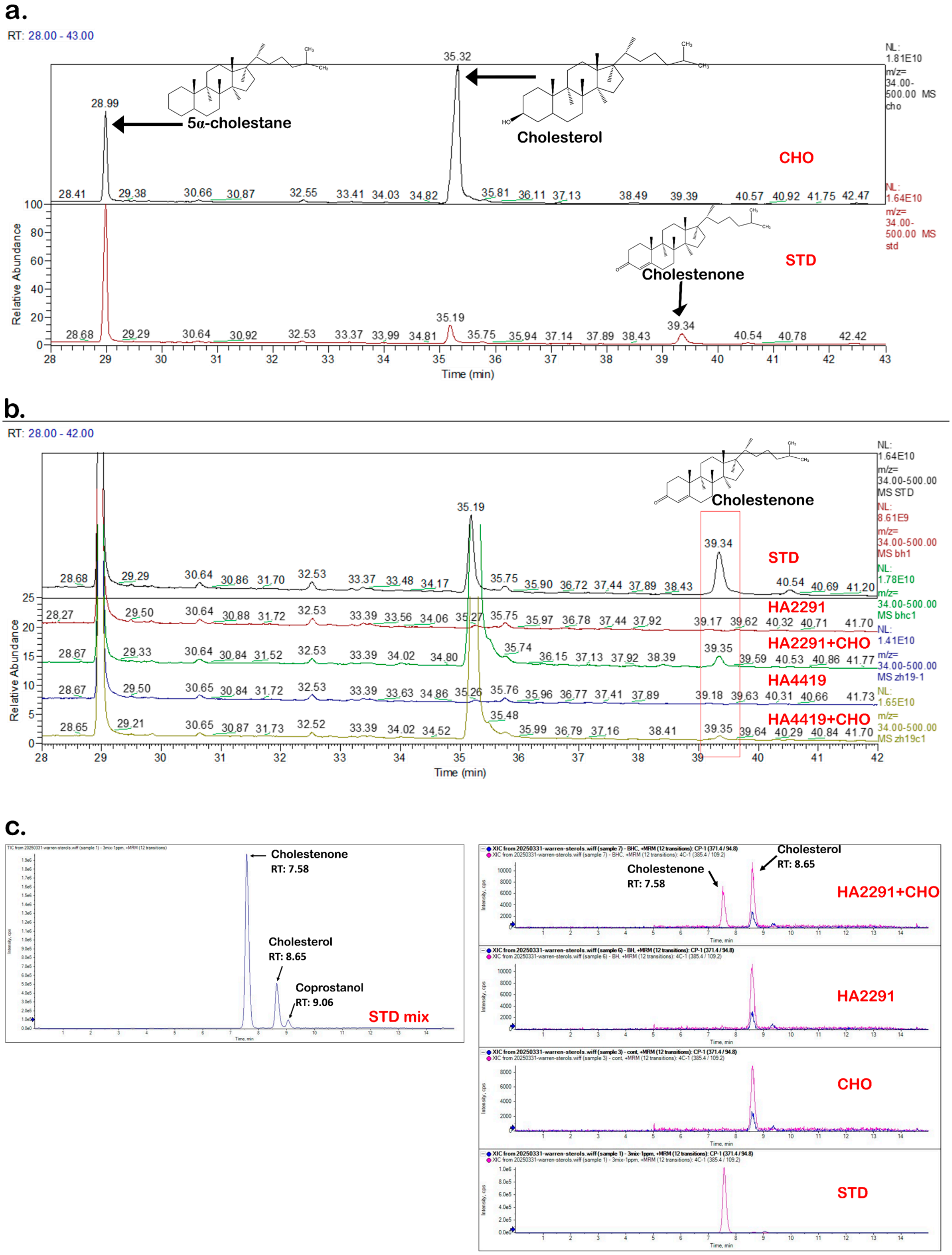

a.
RT: 28.00 - 43.00
NL: 1.81E10 m/z= 34.00-500.00 MS cho
5α-cholestane
28.99
35.32
Cholesterol
CHO
NL: 1.64E10 m/z= 34.00-500.00 MS std
STD
Cholestenone
35.19
39.34
Relative Abundance
Time (min)
b.
RT: 28.00 - 42.00
NL: 1.64E10 m/z= 34.00-500.00 MS STD
NL: 8.61E9 m/z= 34.00-500.00 MS bh1
NL: 1.78E10 m/z= 34.00-500.00 MS bhc1
NL: 1.41E10 m/z= 34.00-500.00 MS zh19-1
NL: 1.65E10 m/z= 34.00-500.00 MS zh19c1
Cholestenone
35.19
39.34
STD
HA2291
HA2291+CHO
HA4419
HA4419+CHO
39.35
Relative Abundance
Time (min)
c.
Cholestenone
RT: 7.58
Cholesterol
RT: 8.65
Coprostanol
RT: 9.06
STD mix
Cholesterol
RT: 8.65
Cholestenone
RT: 7.58
HA2291+CHO
HA2291
CHO
STD

Figure S1. GC-MS and LC-MS chromatograms showing cholestenone production by *B. hominis* HA2291 grown with cholesterol. (**a**) GC-MS chromatograms of chemical standards (STD) and uninoculated cholesterol control (CHO). (**b**) GC-MS chromatograms of *B. hominis* HA2291 grown with cholesterol (HA2291+CHO) and without cholesterol (HA2291), alongside STD and CHO controls. The red frame highlights the presence or absence of peaks corresponding to cholestenone (cholest-4-en-3-one). (**c**) LC-MS chromatograms of *B. hominis* HA2291 showing peaks corresponding to cholestenone and cholesterol.

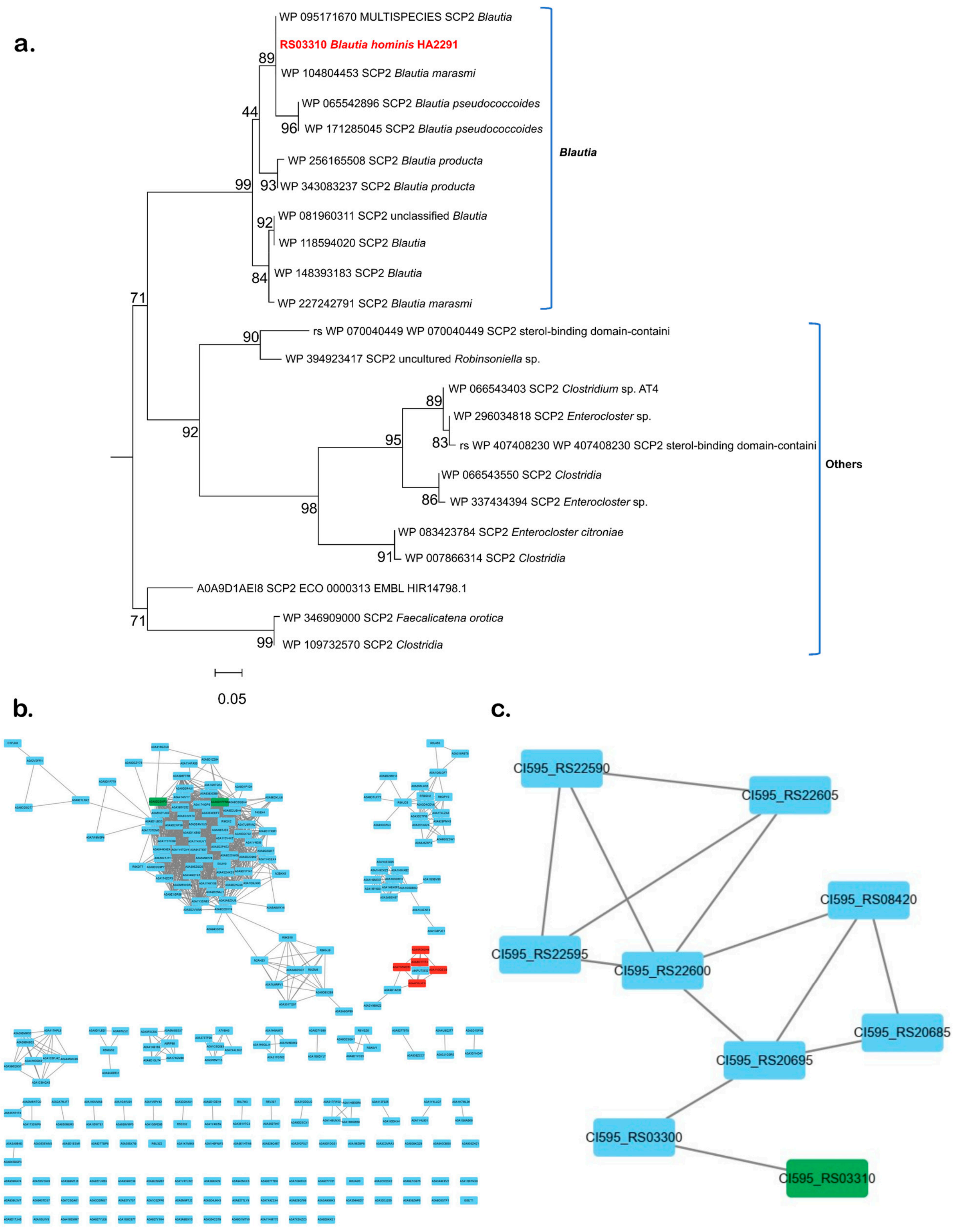
a.
WP 095171670 MULTISPECIES SCP2 Blautia
RS03310 Blautia hominis HA2291
WP 104804453 SCP2 Blautia marasmi
WP 065542896 SCP2 Blautia pseudococcoides
WP 171285045 SCP2 Blautia pseudococcoides
WP 256165508 SCP2 Blautia producta
WP 343083237 SCP2 Blautia producta
WP 081960311 SCP2 unclassified Blautia
WP 118594020 SCP2 Blautia
WP 148393183 SCP2 Blautia
WP 227242791 SCP2 Blautia marasmi
Blautia
rs WP 070040449 WP 070040449 SCP2 sterol-binding domain-containi
WP 394923417 SCP2 uncultured Robinsoniella sp.
WP 066543403 SCP2 Clostridium sp. AT4
WP 296034818 SCP2 Enterocloster sp.
rs WP 407408230 WP 407408230 SCP2 sterol-binding domain-containi
WP 066543550 SCP2 Clostridia
WP 337434394 SCP2 Enterocloster sp.
WP 083423784 SCP2 Enterocloster citroniae
WP 007866314 SCP2 Clostridia
A0A9D1AEI8 SCP2 ECO 0000313 EMBL HIR14798.1
WP 346909000 SCP2 Faecalicatena orotica
WP 109732570 SCP2 Clostridia
Others
89
44
96
99
93
92
84
71
90
89
95
83
92
86
98
91
71
99
0.05
b.
c.
CI595_RS22590
CI595_RS22605
CI595_RS08420
CI595_RS22595
CI595_RS22600
CI595_RS20685
CI595_RS20695
CI595_RS03300
CI595_RS03310

Figure S2. In silico analyses positioning RS03310 as a sterol carrier protein 2 (SCP2) domain-containing protein in *B. hominis* HA2291. (**a**) Maximum likelihood phylogenetic tree of bacterial SCP2 domains, with RS03310 highlighted in red. The *Blautia* clade (Shimodaira-Hasegawa approximate likelihood ratio test (SH-aLRT) support: 99 %) clusters distinctly from other bacterial clades (SH-aLRT = 92 %, and 71 % support). Scale bar: expected substitutions per site (PhyML analysis, visualized in MEGA12). (**b**) Sequence similarity network (SSN) of RS03310, with nodes for *Blautia* (red), Candidatus *Blautia* (green), and other taxa (sky blue). (**c**) Predicted protein-protein interaction network for RS03310 (green) with other *B. hominis* HA2291 proteins (light blue).

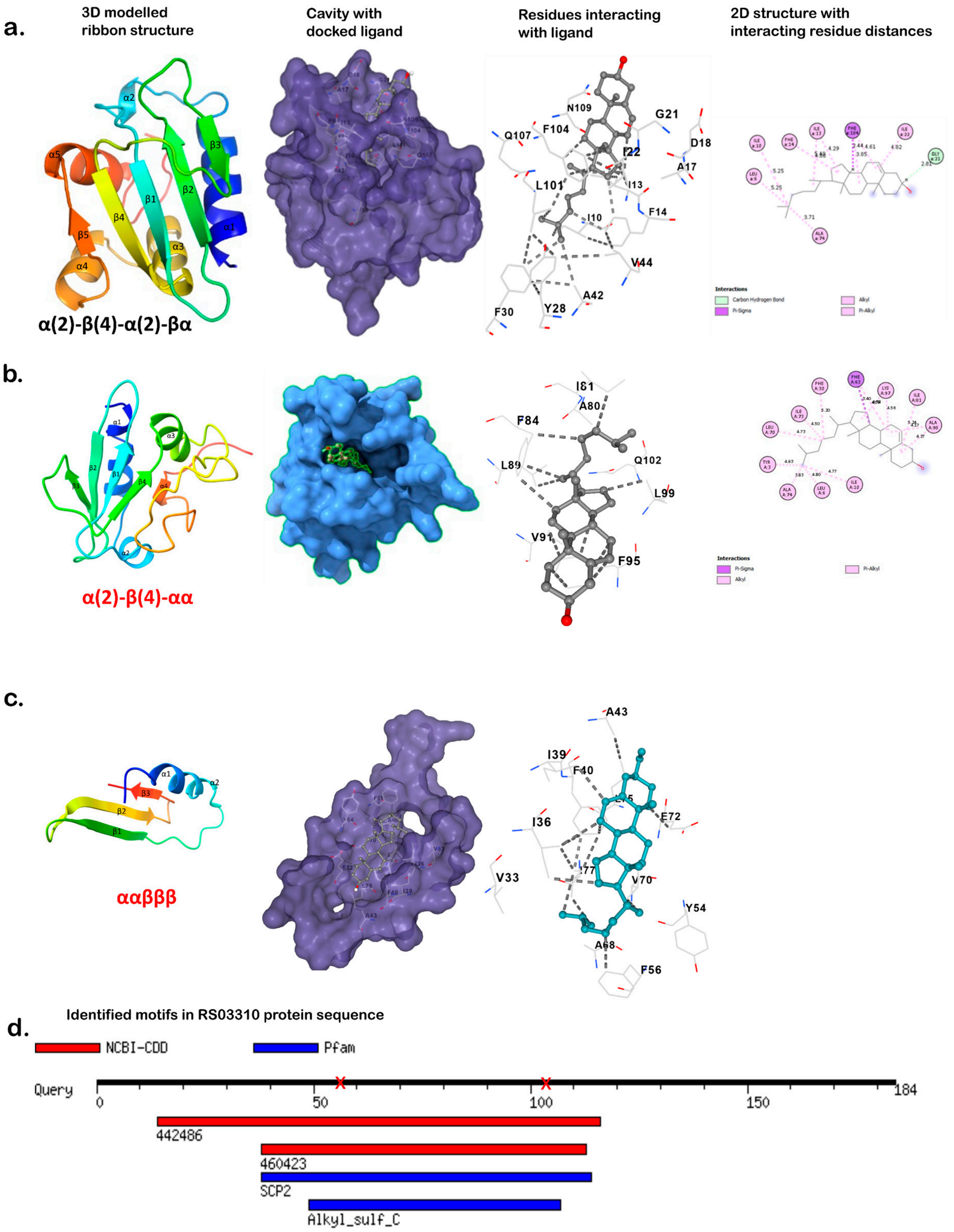

442486: COG3255, SCP2, putative sterol carrier protein, contains SCP2 domain (position: 14..116, Score:66.1, Evalue: 1e14)

460423: pfam02036, SCP2, SCP2 sterol transfer family (position: 38..113, Score: 52.3, Evalue: 2e-09)

SCP2: PF02036, SCP2, SCP2 sterol transfer family (position: 38..114, Evalue: 5.3e-10)

Alkyl_sulf_C: PF14864, Alkyl sulfatase C-terminal (position: 49..107, Evalue: 0.092)

Figure S3. Cholesterol docking and motif analysis of RS03310. (**a**) Predicted 3D ribbon and surface structure of wild-type RS03310 with the best cholesterol-binding cavity identified using CB-Dock2. Cholesterol and interacting amino acid residues in the binding pocket are shown without the surface overlay. A 2D interaction diagram illustrates key residues and intermolecular distances (Å, angstrom). (**b**) Mutated RS03310 model with substitutions F104-N109 → S104-K109 and point mutations G21C and I22T. (c) RS03310 protein truncated at the C-terminal region of the predicted SCP2 motif. (**d**) Conserved motifs within the RS03310 sequence; red "X" marks indicate the deleted region shown in panel (**c**). Motif analysis was performed using MOTIF Search (https://www.genome.jp/tools/motif/; accessed February 15, 2025).

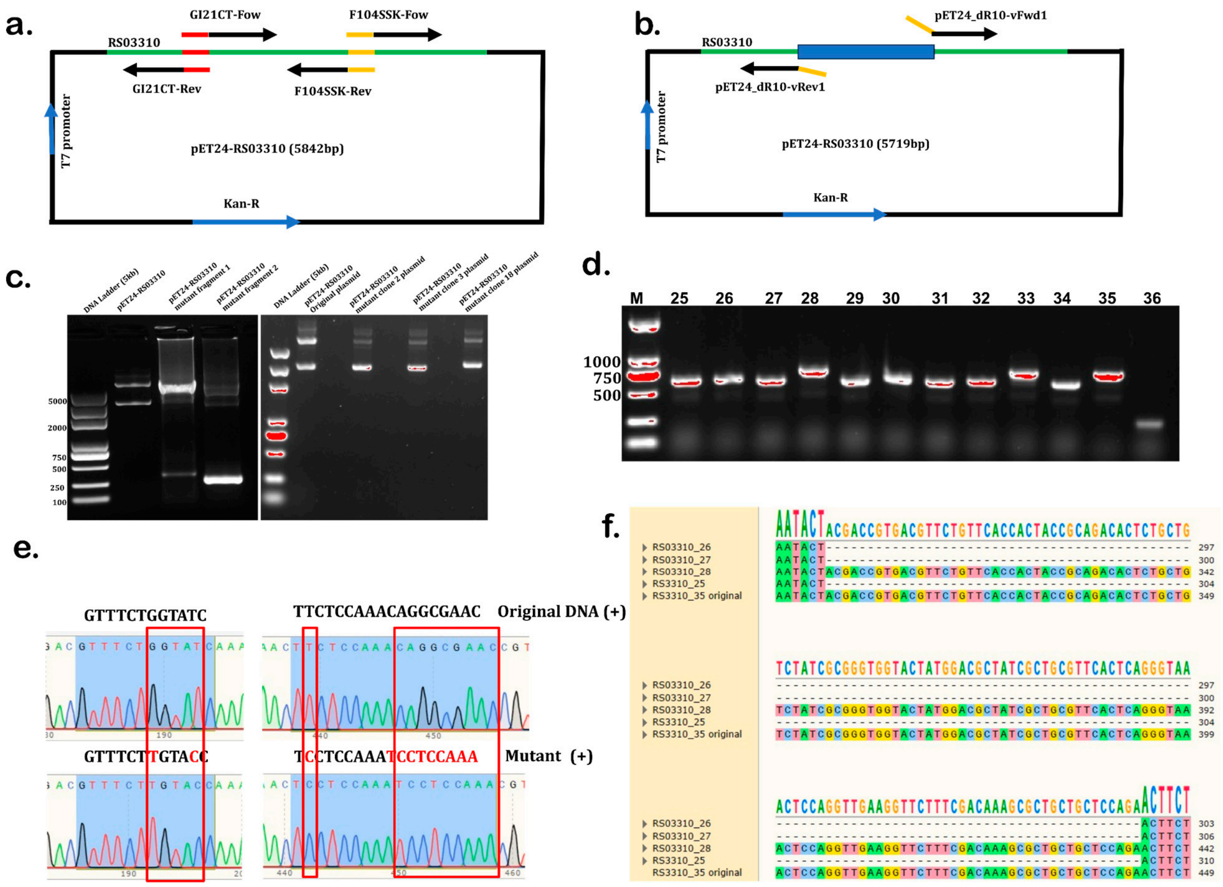

Figure S4. Vector-based site-directed mutagenesis of RS03310 to generate a double mutant and a partial deletion. (**a**) Schematic representation of site-directed mutagenesis to generate mRS03310 by overlap extension PCR, introducing a double mutation at positions 104SSKSSK109 (brown) and 21CT22 (red) within the RS03310 gene insert (green). (**b**) Schematic of a partial deletion on the C-terminal side of the RS03310 gene insert (blue region) to generate dRS03310. (**c**) Agarose gel electrophoresis showing the uncut plasmid and two PCR fragments amplified using primers F104SSK-Fow/GI21CT-Rev (fragment 1, 5584 bp) and GI21CT-Fow/F104SSK-Rev (fragment 2, 267 bp), as well as colony PCR screening of successful mRS03310 clones. (**d**) Agarose gel electrophoresis showing colony PCR products of dRS03310 clones with the expected deletion (bands present in all lanes except 28, 33, and 35 [original sequence] and 36 [negative control]). (**e**) Sanger sequencing chromatograms confirming base substitutions in mRS03310 relative to the original clone (highlighted in red frames). (**f**) Alignment of sequences showing the deleted region in dRS03310 relative to the original clone (lane 35), visualized using SnapGene v6.0.2.

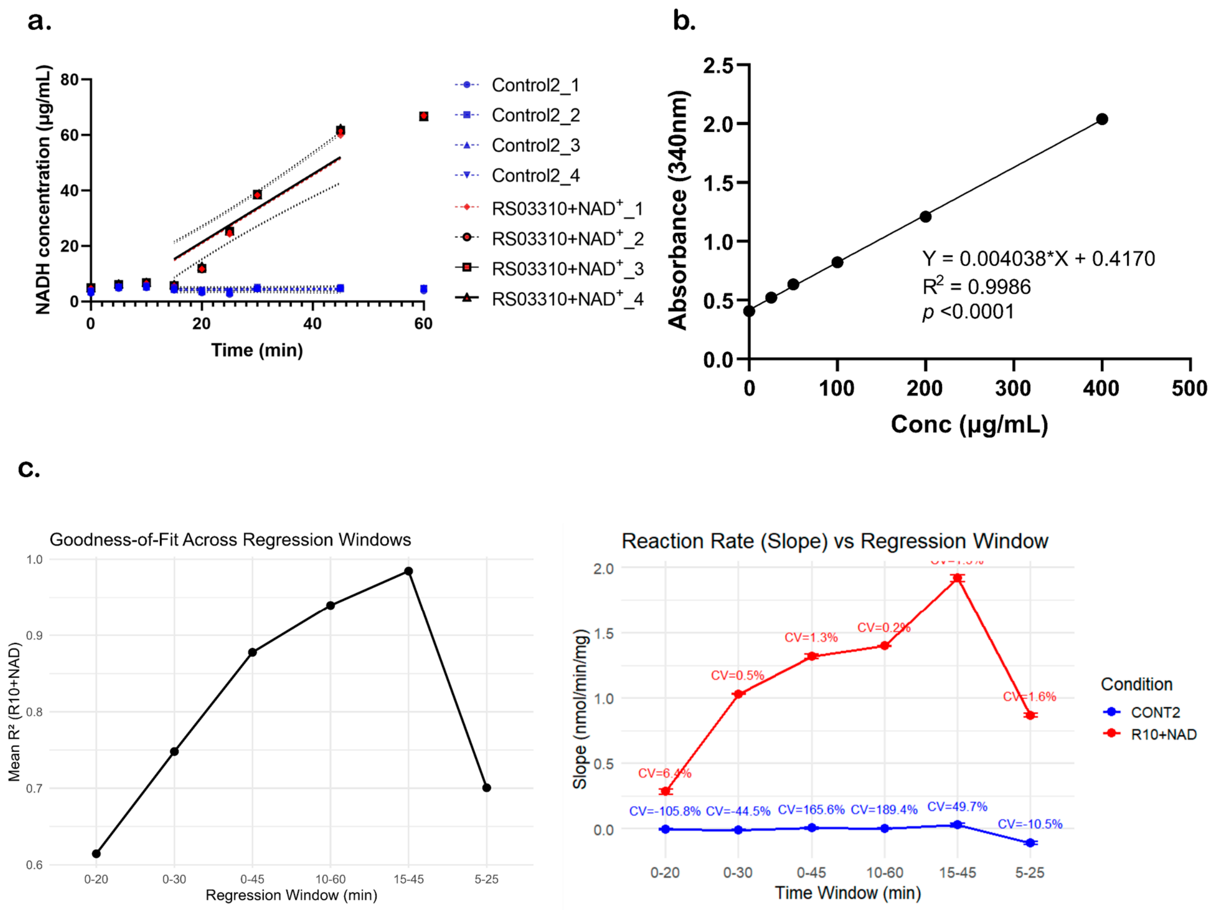

Figure S5. Linear regression-based time-window selection for enzymatic kinetic analysis (**a**) Time-dependent NADH production by RS03310. RS03310wt (RS03310 + NAD^+; red symbols) catalyzed NADH formation in a time-dependent manner, whereas control reactions (Control2; blue symbols) showed no measurable increase in NADH concentration over the same period. Data points represent independent biological replicates (n = 4 per condition, as described in Methods). Linear regression was performed within the empirically selected linear phase (15-45 min; dashed lines). RS03310^wt reactions exhibited strong linearity (mean R^2 = 0.92-0.93), whereas control reactions showed negligible correlation (R^2 = 0.00-0.02), consistent with background signal. Slopes derived from the selected linear window were used to calculate specific activity and to establish the assay limit of detection, as detailed in the Methods and Table S8 (**b**) NADH calibration curve measured at A_{340}. (**c**) Goodness-of-fit metrics (mean R^2 vs candidate time windows) and regression-derived rate estimates across candidate time windows used to determine the optimal 15-45 min interval for linear rate estimation.

Table S1. The LC mass transitions

**Compound name**	**Q1 mass (amu)**	**Q3 mass (amu)**	**DP(V)**	**CE(V)**
Cholesterol_Quantifier	369.400	161.200	100.00	30.00
Cholesterol_Qualifier1	369.400	147.100	100.00	35.00
Cholesterol_Qualifier2	369.400	135.100	100.00	29.00
Coprostanol_Quantifier	371.400	94.800	100.00	46.00
Coprostanol_Qualifier1	371.400	109.100	100.00	38.00
Coprostanol_Qualifier2	371.400	149.200	100.00	29.00
Cholestenone_Quantifier	385.400	109.200	100.00	42.00
Cholestenone_Qualifier1	385.400	97.200	100.00	38.00
Cholestenone_Qualifier2	385.400	367.400	100.00	26.00

CE, collision energies; DP, declustering potentials

Table S2. Linear regression-based time-window selection for enzymatic kinetic analysis. Comparison of candidate time intervals used for linear regression-based estimation of enzymatic reaction rates. For each tested interval, goodness-of-fit metrics including R^2, adjusted R^2, and residual standard error (RSE) were calculated across biological replicates. The 15-45 min interval demonstrated optimal linearity (mean R^2 = 0.98), minimal residual error, and stable slope estimates, and was therefore selected for specific activity calculation.

**Window**	**Condition**	**Mean_Slope**	**SD_Slope**	**CV**	**Mean_R²**	**Mean_Adj_R²**	**Mean_Residual_SE**
0-20	CONT2	-0.01	0.01	-105.79	0.01	-0.32	0.95
0-30	CONT2	-0.01	0.00	-44.46	0.02	-0.17	0.91
0-45	CONT2	0.00	0.01	165.56	0.01	-0.15	0.87
10-60	CONT2	0.00	0.00	189.39	0.01	-0.19	0.82
15-45	CONT2	0.03	0.01	49.73	0.20	-0.07	0.77
5-25	CONT2	-0.11	0.01	-10.50	0.88	0.84	0.36
0-20	R10+NAD	0.28	0.02	6.38	0.61	0.49	2.04
0-30	R10+NAD	1.03	0.00	0.48	0.75	0.70	7.07
0-45	R10+NAD	1.32	0.02	1.30	0.88	0.86	7.75
10-60	R10+NAD	1.40	0.00	0.24	0.94	0.93	6.85
15-45	R10+NAD	1.92	0.03	1.49	0.98	0.98	3.18
5-25	R10+NAD	0.87	0.01	1.63	0.70	0.60	5.17

Table S6. Summary of sample sequencing data quality for BH (non-cholesterol environment) and BHC (in cholesterol environment). Q20 and Q30 represent the percentage of bases with Phred value N20 and N30, respectively

Sample name	Raw reads	Clean reads	Raw bases	Clean bases	Error rate (%)	Q20 (%)	Q30 (%)	GC content (%)
**BH_1**	7910570	7744264	1.2G	1.2G	0.03	97.48	92.96	46.05
**BH_2**	7778562	7684098	1.2G	1.2G	0.03	97.36	92.61	44.98
**BH_3**	6535522	6400814	1.0G	1.0G	0.03	96.94	91.78	46.43
**BHC_1**	7664302	7532398	1.1G	1.1G	0.03	96.69	91.66	46.08
**BHC_2**	8112314	7805056	1.2G	1.2G	0.03	97.37	92.68	46.02
**BHC_3**	7819378	7521344	1.2G	1.1G	0.03	97.32	92.57	46.25

Table S7. Statistics on the alignment of samples with the reference genome of *Blautia producta* (ncbi_blautia_gcf_002270465_1_asm227046v1)

Sample name	BH_1	BH_2	BH_3	BHC_1	BHC_2	BHC_3
**Total reads**	7744264	7684098	6400814	7532398	7805056	7521344
**Total mapped reads**	6633326	6558520	5463873	6569111	6953375	6831165
**Uniquely mapped reads**	6264360	6259707	5269597	6172288	6721318	6616123
**Multiple mapped reads**	368966	298813	194276	396823	232057	215042
**Total mapping rate**	85.65%	85.35%	85.36%	87.21%	89.09%	90.82%
**Uniquely mapping rate**	80.89%	81.46%	82.33%	81.94%	86.11%	87.96%
**Multiple mapping rate**	4.76%	3.89%	3.04%	5.27%	2.97%	2.86%

Table S9. Primers for vector-based site directed mutagenesis for pET24-RS03310

Primer name	Sequence (5'-3')	Expected mutation
F104SSK-Fow	TCCTCCAAATCCTCCAAACGTGAGAAGAAGAAACAGAAGAAAGCGG	104SSKSSK109
F104SSK-Rev	TTTGGAGGATTTGGAGGAGTTCTGGAGCAGCAGC	104SSKSSK109
GI21CT-Fow	GACGTTTCTTGTACCAAAGAACACATCGCGTACCAG	21CT22
GI21CT-Rev	TTTGGTACAAGAAACGTCAGCCTGCATGAAGATGTCAC	21CT22
pET24_dR10_vRev1	CGCCTGTTTGGAGAAGTAGTATTCGTACGGTTCGATGTACA	Partial deletion
pET24_dR10_vFwd1	TTCTCCAAACAGGCGAACCGTGAGAA	Partial deletion
qPCR analysis		
RS03310-F	CGGATGTGAGCGGGATCAAGG	Expected size = 181bp
RS03310-R	GTGCCTCCCGCAATGCTCAA	
16S-F	GACATCCCTCTGACGTGCC	109bp
16S-R	TGCGGGACTTAACCCAACAT	

References

1. Langmead, B.; Salzberg, S.L. Fast gapped-read alignment with Bowtie 2. *Nat Methods* **2012**, *9*, 357-359, doi:10.1038/nmeth.1923.
2. Anders, S.; Pyl, P.T.; Huber, W. HTSeq—a Python framework to work with high-throughput sequencing data. *Bioinformatics* **2014**, *31*, 166-169, doi:10.1093/bioinformatics/btu638.
3. Zhao, S.; Ye, Z.; Stanton, R. Misuse of RPKM or TPM normalization when comparing across samples and sequencing protocols. *Rna* **2020**, *26*, 903-909, doi:10.1261/rna.074922.120.
4. Wang, L.; Feng, Z.; Wang, X.; Wang, X.; Zhang, X. DEGseq: an R package for identifying differentially expressed genes from RNA-seq data. *Bioinformatics* **2010**, *26*, 136-138, doi:10.1093/bioinformatics/btp612.
5. Haynes, W. Benjamini–Hochberg Method. In *Encyclopedia of Systems Biology*, Dubitzky, W., Wolkenhauer, O., Cho, K.-H., Yokota, H., Eds.; Springer New York: New York, NY, 2013; pp. 78-78.
6. Zallot, R.; Oberg, N.; Gerlt, J.A. The EFI Web Resource for Genomic Enzymology Tools: Leveraging Protein, Genome, and Metagenome Databases to Discover Novel Enzymes and Metabolic Pathways. *Biochemistry* **2019**, *58*, 4169-4182, doi:10.1021/acs.biochem.9b00735.
7. Oberg, N.; Zallot, R.; Gerlt, J.A. EFI-EST, EFI-GNT, and EFI-CGFP: Enzyme Function Initiative (EFI) Web Resource for Genomic Enzymology Tools. *Journal of Molecular Biology* **2023**, *435*, 168018, doi:https://doi.org/10.1016/j.jmb.2023.168018.
8. Shannon, P.; Markiel, A.; Ozier, O.; Baliga, N.S.; Wang, J.T.; Ramage, D.; Amin, N.; Schwikowski, B.; Ideker, T. Cytoscape: a software environment for integrated models of biomolecular interaction networks. *Genome Res* **2003**, *13*, 2498-2504, doi:10.1101/gr.1239303.
9. Dereeper, A.; Guignon, V.; Blanc, G.; Audic, S.; Buffet, S.; Chevenet, F.; Dufayard, J.F.; Guindon, S.; Lefort, V.; Lescot, M.; et al. Phylogeny.fr: robust phylogenetic analysis for the non-specialist. *Nucleic Acids Res* **2008**, *36*, W465-469, doi:10.1093/nar/gkn180.
10. Breitsprecher, D.; Fung, P.A.; Tschammer, N. Improving biosensor assay development by determining sample quality with Tycho NT.6. *Nature Methods* **2018**, *15*, 298-298, doi:10.1038/nmeth.f.406.
11. Powell, H.R.; Islam, S.A.; David, A.; Sternberg, M.J.E. Phyre2.2: A Community Resource for Template-based Protein Structure Prediction. *Journal of Molecular Biology* **2025**, 168960, doi:https://doi.org/10.1016/j.jmb.2025.168960.
12. Choinowski, T.; Hauser, H.; Piontek, K. Structure of sterol carrier protein 2 at 1.8 A resolution reveals a hydrophobic tunnel suitable for lipid binding. *Biochemistry* **2000**, *39*, 1897-1902, doi:10.1021/bi992742e.
13. Pettersen, E.F.; Goddard, T.D.; Huang, C.C.; Meng, E.C.; Couch, G.S.; Croll, T.I.; Morris, J.H.; Ferrin, T.E. UCSF ChimeraX: Structure visualization for researchers, educators, and developers. *Protein Sci* **2021**, *30*, 70-82, doi:10.1002/pro.3943.

14. Meng, E.C.; Goddard, T.D.; Pettersen, E.F.; Couch, G.S.; Pearson, Z.J.; Morris, J.H.; Ferrin, T.E. UCSF ChimeraX: Tools for structure building and analysis. *Protein Sci* **2023**, *32*, e4792, doi:10.1002/pro.4792.
15. Hanwell, M.D.; Curtis, D.E.; Lonie, D.C.; Vandermeersch, T.; Zurek, E.; Hutchison, G.R. Avogadro: an advanced semantic chemical editor, visualization, and analysis platform. *Journal of Cheminformatics* **2012**, *4*, 17, doi:10.1186/1758-2946-4-17.
16. Yang, X.; Liu, Y.; Gan, J.; Xiao, Z.-X.; Cao, Y. FitDock: protein–ligand docking by template fitting. *Briefings in Bioinformatics* **2022**, *23*, doi:10.1093/bib/bbac087.
17. Liu, Y.; Yang, X.; Gan, J.; Chen, S.; Xiao, Z.-X.; Cao, Y. CB-Dock2: improved protein–ligand blind docking by integrating cavity detection, docking and homologous template fitting. *Nucleic Acids Research* **2022**, *50*, W159-W164, doi:10.1093/nar/gkac394.
18. Liu, Y.; Cao, Y. Protein-Ligand Blind Docking Using CB-Dock2. *Methods Mol Biol* **2024**, *2714*, 113-125, doi:10.1007/978-1-0716-3441-7_6.
19. Kelley, L.A.; Sternberg, M.J. Protein structure prediction on the Web: a case study using the Phyre server. *Nat Protoc* **2009**, *4*, 363-371, doi:10.1038/nprot.2009.2.
